# Real-space imaging of the electron-pair density hole in molecular Auger–Meitner decay

**DOI:** 10.1038/s41567-026-03363-8

**Published:** 2026-06-30

**Authors:** Mats Simmermacher, Nathan Goff, Andres Moreno Carrascosa, Elke Fasshauer, Thomas Northey, Lingyu Ma, Haiwang Yong, Brian Stankus, Asami Odate, Xuan Xu, Wenpeng Du, Kyle Acheson, Joseph C. Cooper, Daniel Ratner, Mengning Liang, Ruaridh Forbes, Michael P. Minitti, Adam Kirrander, Peter M. Weber

**Affiliations:** 1 Physical and Theoretical Chemistry Laboratory, Department of Chemistry, University of Oxford, Oxford, UK; 2 Department of Chemistry, Brown University, Providence, RI USA; 3 Institute of Physical and Theoretical Chemistry, University of Tübingen, Tübingen, Germany; 4 Center for Light–Matter Interaction, Sensors and Analytics (LISA⁺), University of Tübingen, Tübingen, Germany; 5 Linac Coherent Light Source, SLAC National Accelerator Laboratory, Menlo Park, CA USA; 6 Present Address: Department of Chemistry, University of California San Diego, La Jolla, CA USA; 7 Present Address: Department of Chemistry and Biochemistry, Western Connecticut State University, Danbury, CT USA; 8 Present Address: School of Chemistry and Chemical Engineering, University of Southampton, Southampton, UK; 9 Present Address: National Research Council Canada, Ottawa, Ontario Canada; 10 Present Address: Thomas Jefferson National Accelerator Facility, Newport News, VA USA; 11 Present Address: Department of Physics, Old Dominion University, Norfolk, VA USA; 12 Present Address: Department of Chemistry, University of California, Davis, Davis, CA USA

**Keywords:** Attosecond science, Atomic and molecular interactions with photons, Chemical physics, Imaging techniques, Molecular dynamics

## Abstract

Electrons in matter can rearrange extremely quickly under external perturbations, underpinning subsequent structural and chemical transformations. Coulomb interactions between neighbouring electrons often shape this response, giving rise to correlated motion and strongly affecting the distribution of electrons in the system. Here we show that non-resonant hard X-ray scattering can directly access changes in the radial electron-pair density during the rapid rearrangement of core and valence electrons. We do this by studying sulfur hexafluoride molecules undergoing Auger–Meitner decay. We exploit a second-order interaction between the X-ray photons and the molecules to trigger and probe the decay dynamics with a single pulse, capturing the electron loss and redistribution before the molecules dissociate. The experiment shows that changes in electron-pair densities can be isolated and measured on ultrafast timescales, providing insight into the real-space evolution of highly excited and short-lived electronic states.

## Abstract (web summary)

Localized core vacancies in molecules decay via an Auger–Meitner cascade to form diffuse valence holes. X-ray scattering is now used to measure the change in the electron-pair density, resolving the spatial redistribution of electrons.

## Main

When matter is irradiated by light or subjected to other external perturbations, the electrons rearrange in response. Because the motion of each electron is coupled to all other electrons by Coulomb repulsion, the collective response of the electronic system constitutes a challenging many-body problem. A striking example of electron dynamics driven by Coulomb interaction is that of Auger–Meitner decay^1–4^, where core-level ionization initiates a rapid cascade of electronic transitions.

**Fig. 1:**
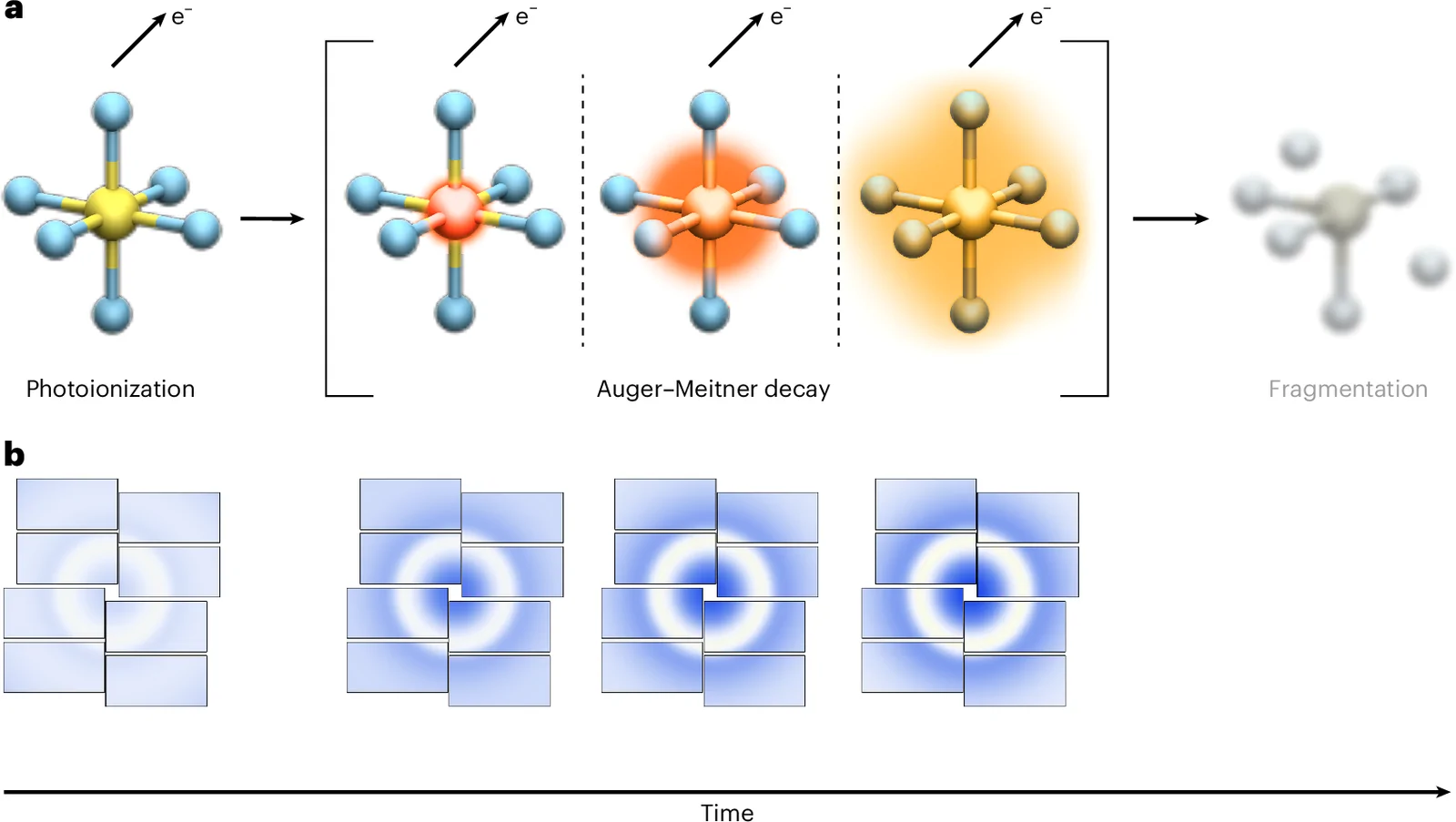
Schematic of the electron dynamics and the non-resonant X-ray scattering signal that probes it. **a**, A photoionized SF_6_ molecule undergoes Auger–Meitner decay within 15 fs. In the depicted channel, initial photoionization creates a vacancy in the K shell of the central sulfur atom. Next, this vacancy decays into a double vacancy in the L shell, whereby a second electron is ejected. In the second Auger-Meitner step, a vacancy in the L shell remains and a double vacancy in the molecule’s valence is formed, again by ejecting an electron. The decay channel terminates with the creation of a quadruple vacancy in the valence. The simulated population dynamics of that channel are shown in Extended Data Fig. 1. Further details on all dominant decay channels for photoionization of both the sulfur and the fluorine atoms are given in Supplementary Section 5.1. At later times not captured in this experiment, the highly charged and destabilized molecular cation undergoes fragmentation due to Coulomb explosion. **b**, Simulated detector images that show the change in the number of scattered X-ray photons on each pixel. Each detector image captures a snapshot of the dynamics illustrated in **a**.

Such electron dynamics are illustrated in Fig. 1 for core-excited sulfur hexafluoride (SF_6_). Initial absorption of an X-ray photon ionizes the molecule and creates an unstable inner-shell vacancy. This vacancy decays via the concerted motion of two energetically higher lying electrons, with one electron filling the vacancy and the other ejected to carry off the excess energy. Thereby, the original vacancy is replaced by two new, less energetic ones. This process repeats until the system runs out of energy for further ionization, increasing the positive charge on the molecule at each step. The initially compact and localized hole in the electron density grows and becomes more diffuse and distributed across the whole molecule. Eventually, the electrostatically destabilized molecule fragments. This type of electron dynamics is important in the context of radiation damage^5–8^ and has been studied in real time by means of attosecond spectroscopy^9–17^, which can identify the states involved in the dynamics in terms of their evolution in energy.

To fully characterize Coulomb-driven electron dynamics, it is desirable to complement spectroscopic data with direct information about the spatial rearrangement of the electrons. We demonstrate here that this can be achieved by ultrafast nonresonant X-ray scattering of isolated molecules in the gas phase^18^. A straightforward transformation of the scattering signal from reciprocal into real space yields the difference in the radial electron-pair density that reveals the spatial redistribution of electrons during Auger–Meitner decay. This information complements spectroscopy and other imaging techniques that may also provide insight into the spatial rearrangement of electrons during molecular Auger–Meitner decay. Such imaging techniques include ultrafast photoelectron momentum imaging, photoelectron or laser-induced electron diffraction, and electron microscopy^19–23^.

**Extended Data Fig. 2:**
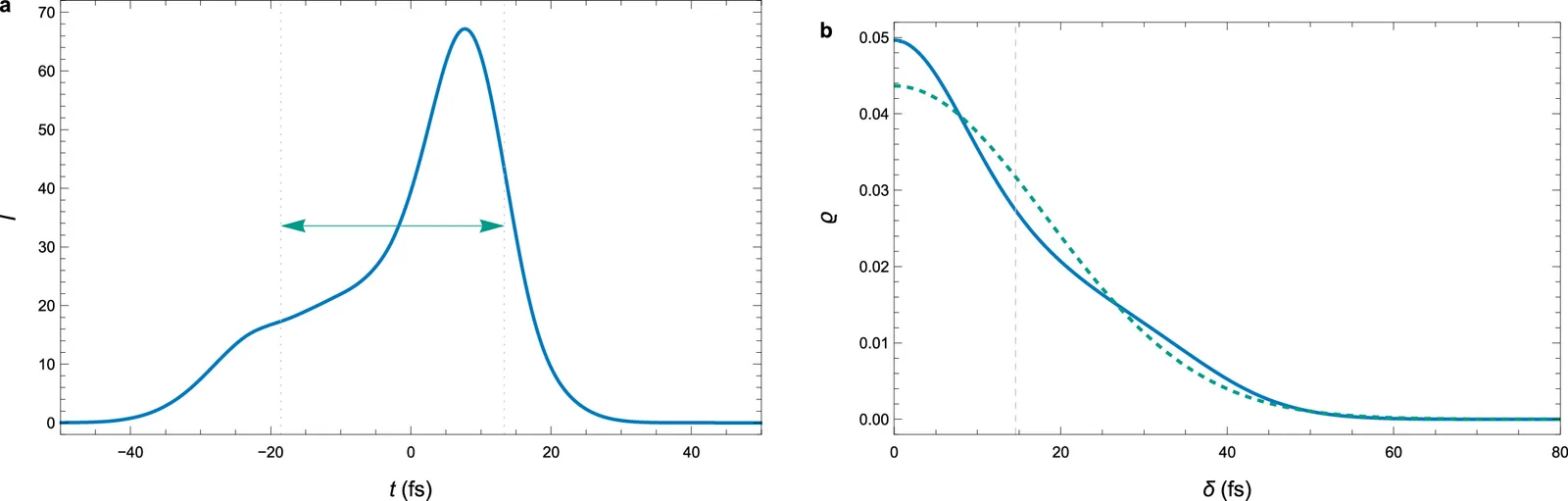
Average intensity-time profile of the incident X-ray pulses and its corresponding distribution of effective pump-probe delay times. **a**) Intensity-time profile, *I*(*t*), measured with the XTCAV and averaged over 145,425 independent shots. The vertical arrow depicts the pulse duration of 31.9 fs, which is defined in analogy to the full-width at half-maximum duration of a Gaussian-shaped pulse. **b**) Distribution of pump-probe delay times, *ϱ*(*δ*), obtained from *I*(*t*) in **a** (solid curve) and for an idealized, Gaussian-shaped envelope (dashed curve). Both distributions have the same average pump-probe delay time of 〈*δ*〉 ≈ 14.6 fs, which is shown as a vertical dashed line. *ϱ*(*δ*) and 〈*δ*〉 are defined in equations S24–S29 of the Supplementary Information.

We are able to realize the measurements with the required sub-15-fs time resolution and currently existing technology by exploiting a second-order interaction, that is, the absorption of one X-ray photon followed by the non-resonant scattering of a second X-ray photon. In this scenario, both photons belong to the same incident pulse and interact with the same molecule. This allows the measurements to be taken with an effective time resolution of approximately half the full-width at half-maximum pulse duration. The average intensity–time profile of the X-ray pulses and its corresponding distribution of pump–probe delay times are shown in Extended Data Fig. 2.

To isolate this second-order interaction, our experiment takes advantage of the inherent intensity fluctuations of X-ray pulses generated by self-amplified spontaneous emission at the Linac Coherent Light Source (LCLS) X-ray free-electron laser. Fluctuations in the number of photons per pulse allow the scattering signal to be measured as a function of intensity. In the average intensity regime of 10^17^–10^18^ W cm^−^^2^ considered here, the sequential absorption and scattering of an X-ray photon give rise to a quadratic term in the number of scattered photons as a function of the number of photons in the incident X-ray pulse. On condition that the population of the molecule’s electronic ground state and the number of incident photons are only weakly depleted by the photoabsorption, the number of photons scattered onto the detector can be expanded as

1 $${n}_{{\rm{s}}}(q,{n}_{0})\,\approx \,{c}_{1}(q)\,{n}_{0}\,+\,{c}_{2}(q)\,{n}_{0}^{2},$$

in analogy to the description of two-photon absorption. The number of scattered photons *n*_s_ in equation (1) is a function of both the magnitude of the momentum transfer, *q*, and the number of incident photons, *n*_0_. The momentum transfer is related to the scattering angle, *θ*_s_, as well as to the mean angular carrier frequency of the X-ray pulse, *ω*_0_, by $q\approx \left(2\,{\omega }_{0}/c\right)\sin ({\theta }_{{\rm{s}}}/2)$, where *c* is the speed of light. Most importantly, the coefficients *c*_1_(*q*) and *c*_2_(*q*) in equation (1) refer to scattering probabilities of the target molecule, the former for the electronic ground state and the latter upon photoionization and subsequent Auger–Meitner decay. A quantitative definition of both coefficients is given in equations (S8)–(S12) in the Supplementary Information.

**Fig. 2:**
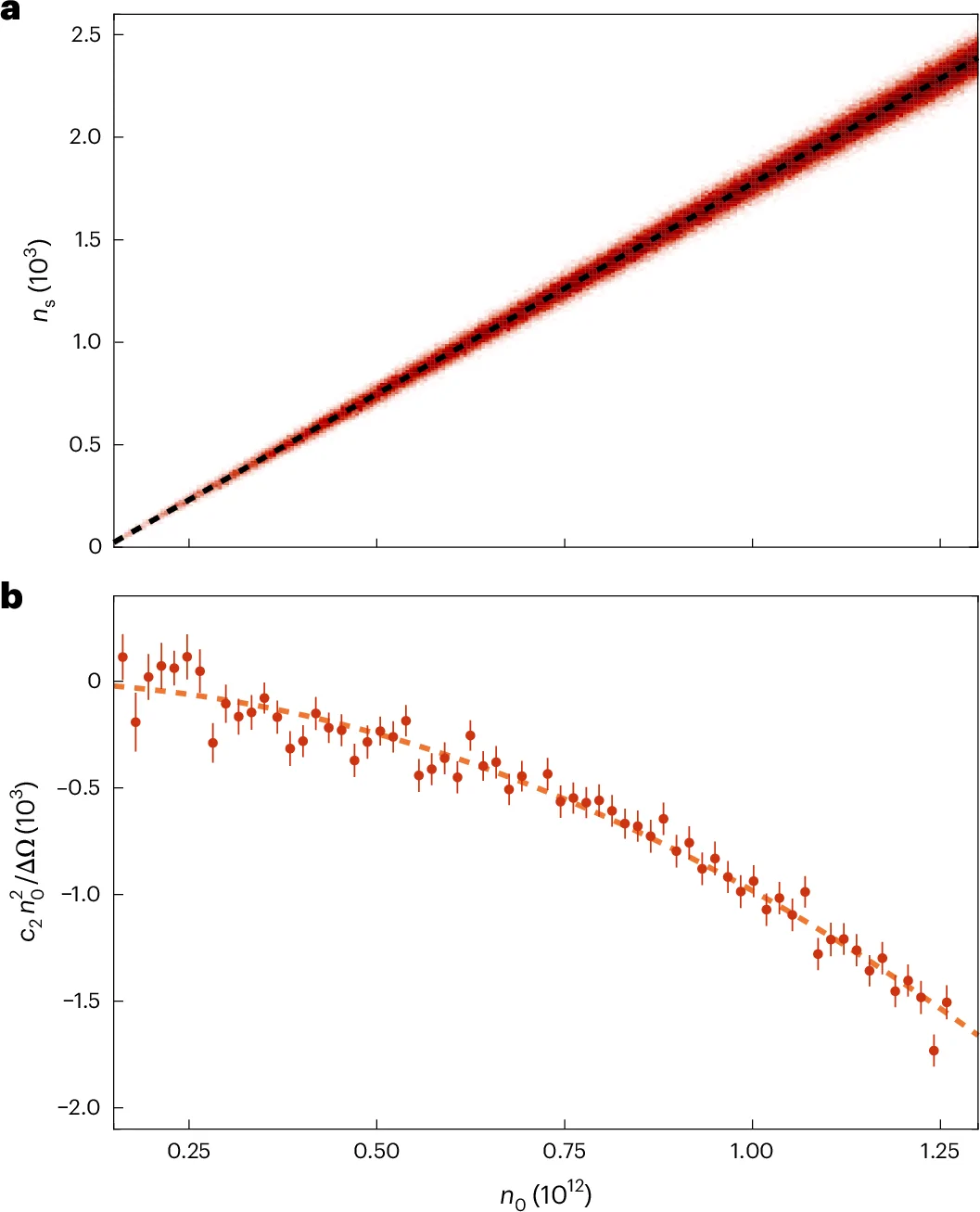
Relationship between the numbers of incident and scattered X-ray photons measured over 171,757 shots with intensities of 10^17^–10^18 ^W cm^−^^2^. **a**, Density histogram of the number shots with *n*_0_ incident and *n*_s_ scattered X-ray photons within the momentum-transfer interval 0.574 Å^−1^ < *q* < 0.677 Å^−1^. **b**, The second-order contribution to the data in **a**. The data points are centres of Gaussians fitted to the respective density histogram in each bin in *n*_0_. The error bars display the 2*σ* (standard deviation) uncertainty normalized by *N*^−1/2^ where *N* ∈ [127, 5,068] is the number of shots recorded within a given bin in *n*_0_. This second-order contribution arises from photoionization by a first and subsequent scattering of a second X-ray photon.

To isolate *c*_1_(*q*) and *c*_2_(*q*), equation (1) is fitted to the measured scattering data. First, the photon counts are binned in *q*. Then, a fit is obtained for each bin to yield the coefficients as functions of *q*. Figure 2a shows how the number of scattered photons relates to the number of incident photons in a specific *q* bin. Figure 2b furthermore displays the second-order contribution obtained by fitting equation (1) to the data in Fig. 2a. This demonstrates that, while the linear component dominates the overall scattering signal, the quadratic component is substantial and can be isolated.

Crucially, the ratio of the quadratic and linear coefficients in equation (1) grants direct access to the relative difference scattering signal,

2 $$\frac{\Delta S(q)}{{S}_{0}(q)}\,=\,\frac{2{\rm{\pi }}{w}^{2}}{{\sigma }_{{\rm{a}}}}\,\frac{{c}_{2}(q)}{{c}_{1}(q)}.$$

Here, *S*_0_(*q*) is the scattering signal of the neutral molecule in its electronic ground state, and Δ*S*(*q*) refers to the difference between the scattering signal of the ionized molecule undergoing Auger–Meitner decay and *S*_0_(*q*). Equation (2) relates Δ*S*(*q*)/*S*_0_(*q*) to the ratio *c*_2_(*q*)/*c*_1_(*q*) via a global scaling factor that depends solely on the beam waist of the X-ray pulse, *w*, and on the photoabsorption cross-section, *σ*_a_, of the SF_6_ molecule. At 9.486 keV mean photon energy, *σ*_a_ is about 4.8 × 10^−21^ cm^2^ and dominated by K-shell ionization from both S(1*s*) and F(1*s*) orbitals^24^. With an overall scaling factor of 1.1 × 10^−13^ deduced from minimizing the mean absolute deviation between the measured and the simulated scattering signals, the beam waist can be estimated to be *w* ≈ 0.91 μm (Supplementary Section 3).

**Fig. 3:**
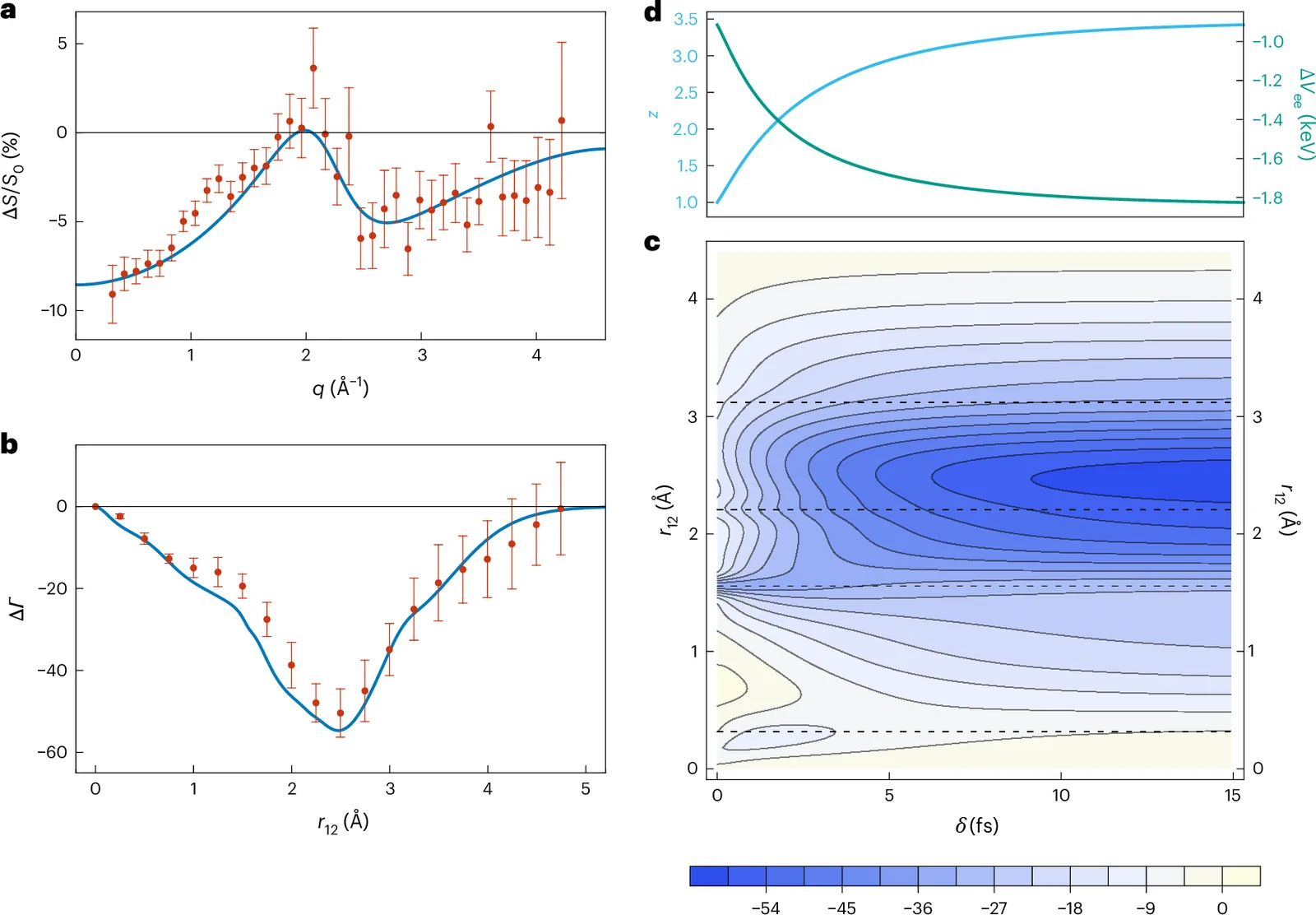
Change in the evolving radial electron-pair density detected by ultrafast X-ray scattering. **a**, Measured (red points) and simulated (blue line) per cent difference scattering signals of core-ionized and Auger–Meitner decaying SF_6_ as a function of momentum transfer *q*. The experimental signal was obtained with 9.486 keV X-ray pulses with an effective average pump–probe delay time of 14.6 fs. It was scaled according to equation (2) such that the mean absolute deviation with respect to the simulated signal was minimized. The error bars display the bootstrapped 1*σ* (standard deviation) uncertainty of the measured signal. The simulation employed a kinetic model and ab initio electronic structure calculations of molecular scattering probabilities. **b**, Pulse-averaged hole in the radial electron-pair density as a function of electron-pair distance *r*_12_, calculated by an inverse spherical Bessel transform of the difference scattering signal Δ*S*(*q*) in **a**. The error bars display the 1*σ* uncertainty derived from bootstrapping within the 3*σ* error of the experimental difference scattering signal. **c**, Simulated evolution of the instantaneous hole in the radial electron-pair density of Auger–Meitner decaying SF_6_ as a function of the electron-pair distance, *r*_12_, and the delay time after the initial K-shell photoionization, *δ*. The colours reflect the depth of the hole. The horizontal dashed lines point out characteristic distances in the molecule. **d**, The molecule’s charge number, *z*, and change in electron-pair repulsion energy, Δ*V*_ee_, corresponding to the simulated dynamics shown in **c**.

**Extended Data Fig. 3:**
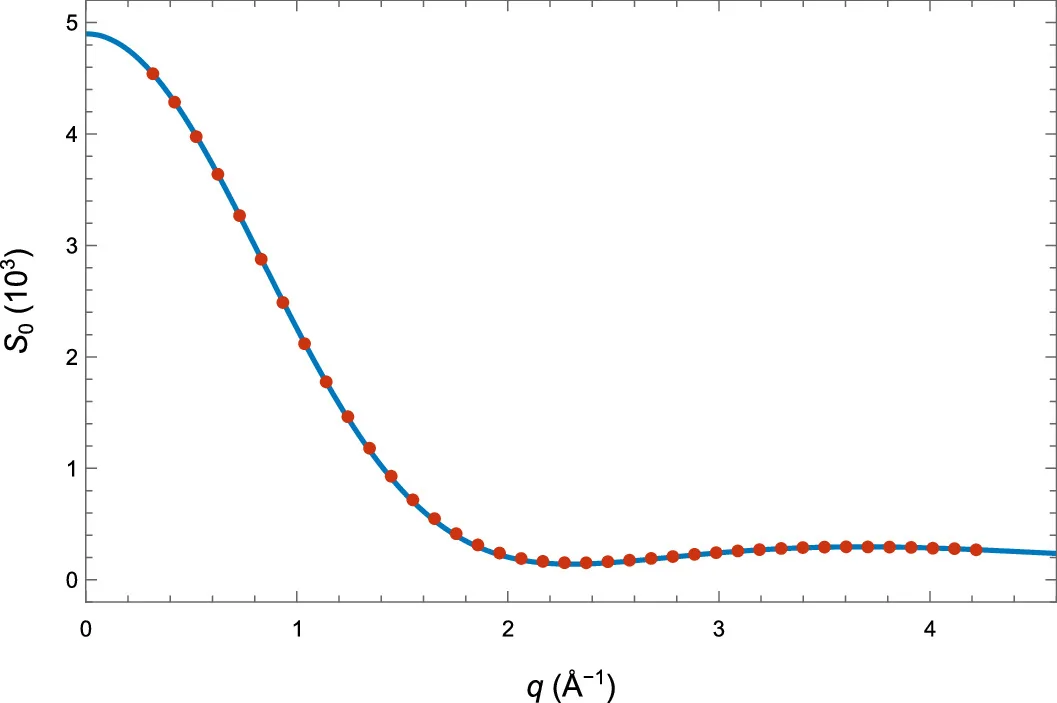
Total isotropic X-ray scattering signal of SF_6_ in its electronic ground state. Shown are the measured (red points) and simulated (blue line) scattering signals as functions of the momentum transfer *q*. They are given in units of the Thomson scattering cross section. The experimental signal was obtained with 9.486 keV X-ray pulses and scaled globally to minimize the mean absolute deviation with respect to the theoretical signal. The bootstrapped 1*σ* uncertainty of the measured signal is visually imperceptible. The simulation employed ab-initio electronic structure theory. Further details are given in section S5.2 of the Supplementary Information.

The relative difference scattering signal Δ*S*(*q*)/*S*_0_(*q*) extracted from the experimental data by means of equations (1) and (2) is shown alongside the theoretical signal in Fig. 3a. Similarly, the measured and simulated ground-state signals *S*_0_(*q*) are displayed in Extended Data Fig. 3. Overall, the measured and simulated curves agree well. Apart from a small, most likely spurious shoulder in the vicinity of *q* ≈ 1.2 Å^−1^ and a few outliers, the simulated scattering signal in Fig. 3a lies consistently within the bootstrapped 1*σ* (standard deviation) confidence interval of the experimental data over the entire range of momentum transfer. It is particularly noteworthy that the maxima in both theory and experiment coincide (at around 2.0 Å^−1^). The same applies to the first local minimum at 2.7 Å^−1^, although the experimental error confines the position less precisely to the interval 2.5–2.9 Å^−1^. The negative signal at low values of *q* reflects the loss of electrons from the molecule, first by photoionization and then by subsequent Auger–Meitner decay. The longer the time between the initial photoionization and the scattering of the second photon, the further the decay proceeds and the stronger the drop of Δ*S*(*q*)/*S*_0_(*q*) at low values of *q*. Here, the signal approaches −8.6% as *q* → 0, revealing that, on average, ~3.1 electrons are ejected from the molecule before it is probed via the scattering of the second hard X-ray photon.

Importantly, the shape of the scattering curve provides detailed information on the electronic structure at the time of scattering. The total isotropic X-ray scattering signal of a molecule in the gas phase probes the radial electron-pair density, *Γ*(*r*_12_), also known as the radial intracule density^25–29^. This density conveys the probability of finding two electrons within the molecule at a distance *r*_12_ from each other. Strikingly, the pulse-averaged change in *Γ*(*r*_12_) for the molecule ionized by the first X-ray photon can be obtained from the absolute difference scattering signal Δ*S*(*q*), which is, except for a few multiplicative parameters, identical to the second-order coefficient *c*_2_(*q*) in equations (1) and (2). The change in *Γ*(*r*_12_) follows directly from Δ*S*(*q*) via an inverse zeroth-order spherical Bessel transform from reciprocal into real space,

3 $$\Delta \varGamma ({r}_{12})\,=\,\frac{{r}_{12}}{{\rm{\pi }}}\,{\int }_{\!\!0}^{\infty }q\,\sin (q{r}_{12})\,\left(\Delta S(q)+\Delta {N}_{{\rm{e}}}\right)\,{\rm{d}}q,$$

where Δ*S*(*q*) is defined in units of the Thomson scattering cross section and Δ*N*_e_ refers to the total number of photoelectrons and Auger–Meitner electrons that move out of the interaction region before the X-ray photon is scattered. We note that, while the value of Δ*N*_e_ cannot be immediately inferred from the experimental scattering data, its overall effect upon Δ*Γ*(*r*_12_) is small. From our model and the experimental parameters, we can deduce that, for the case at hand, Δ*N*_e_ is at most 0.24, compared with a Δ*S*(*q*) of nearly −420 at low *q*. Setting Δ*N*_e_ to zero would affect Δ*Γ*(*r*_12_) by less than 1%, which is well below the experimental uncertainty.

We further note that equation (3) implies that the X-ray scattering signal also contains information about the change in the Coulomb repulsion energy of the electrons in the target^30,31^. This change can in principle be obtained from the difference scattering signal by integration over *q*,

4 $$\Delta {V}_{\mathrm{ee}}\,=\,\frac{1}{{\rm{\pi }}}\,{\int }_{\!\!0}^{\infty }\left(\Delta S(q)+\Delta {N}_{{\rm{e}}}\right)\,{\rm{d}}q,$$

with Δ*V*_ee_ and *q* given in atomic units. The extraction of quantitative values of Δ*V*_ee_ from experimental scattering data, however, requires not only an excellent signal-to-noise ratio and an accurate estimate of the scaling factor but also measurements over a wider range of *q* than in the present experiment. The lack of scattering signal below 0.3 Å^−1^ where Δ*S*(*q*) is strong leads to an underestimation of the decrease in Coulomb repulsion energy by about 60%. While integration of the simulated scattered signal from 0 to 150 Å^−1^ yields Δ*V*_ee_ ≈ −1.72 keV, the value directly extracted from the measured data amounts to only −1.05 ± 0.08 keV, with the given error reflecting solely the bootstrapped 1*σ* uncertainty in the data. Nevertheless, if the simulated signal is restricted to the momentum-transfer range covered by the experiment, the predicted value reduces to −1.02 keV, which is in excellent agreement with the experiment. Although not quantitative, the experimental Δ*V*_ee_ thus clearly reveals that the Coulomb repulsion energy decreases measurably in response to photoionization and demonstrates that non-resonant gas-phase X-ray scattering can grant access to fundamental properties of molecular electronic structure.

The lack of signal at low momentum transfer poses much less of a problem for the radial electron-pair density than for the Coulomb repulsion energy. The factor $q\,\sin (q{r}_{12})$ in equation (3) severely dampens the effect of Δ*S*(*q*) on Δ*Γ*(*r*_12_) at low *q*. The values of Δ*Γ*(*r*_12_) obtained from a straightforward numerical transform of the measured difference scattering signal are shown in Fig. 3b. Despite the limited momentum-transfer range in the experiment, the empirical and simulated data agree remarkably well. Apart from a small deviation in the range 1.0 Å < *r*_12_ < 2.0 Å, the theoretical difference pair density lies consistently within the bootstrapped 1*σ* confidence interval of the experimental data over the entire range of electron–electron distances. The data reveal a broad hole in the radial electron-pair density, which directly reflects the molecule’s loss of electrons due to photoionization and Auger–Meitner decay.

To place the measured electron-pair density hole in Fig. 3b more explicitly into the context of the full decay dynamics, we have simulated the time evolution of the target system for the first 15 fs after photoionization using a kinetic model and ab initio electronic structure calculations. The results of this simulation are shown in Fig. 3c, with Fig. 3d displaying the corresponding increase in the molecule’s charge number, *z*(*δ*), and the decrease in Coulomb repulsion energy, Δ*V*_ee_(*δ*). While *z*(*δ*) and Δ*V*_ee_(*δ*) follow relatively simple exponential decays that reflect the overall relaxation dynamics, the temporally resolved changes in Δ*Γ*(*r*_12_) provide more specific information on how the Auger–Meitner decay proceeds in real space.

During the first 3 fs after photoionization, the electron-pair difference density displays three sharply peaked local minima at around 0.16 Å, 1.56 Å and 2.21 Å as well as a weaker shoulder at 3.12 Å. The presence of these minima reveals that the hole is compact and localized within the core shells of the atoms. The minimum at *r*_12_ ≈ 0.16 Å relates to the distance between a vacancy in either the S(1*s*), F(1*s*) or S(2*s*) orbital and electrons in the same atom, in particular in the same shell. Similarly, the minimum at 1.56 Å correlates with the length of the sulfur–fluorine bond and thus reflects the depletion of electrons in the core of the sulfur and the fluorine atoms. Finally, the minimum at 2.21 Å and the shoulder at 3.12 Å correlate with the distances between two neighbouring and two opposing fluorine atoms, respectively. Over time, these local minima disappear. The hole in the electron-pair density loses structure, deepens and broadens, and moves towards larger values of *r*_12_, with a final minimum at around 2.50 Å. The scattering signal can thus resolve, via the electron-pair density, how the hole expands and migrates from the core into the valence during the Auger–Meitner decay.

In the experimental data shown in Fig. 3a,b, the hole has already dissipated into the valence and we therefore see a rather broad and deep dip in the pair density without further features. Future time-resolved measurements that exploit attosecond X-ray pulses currently developed^32–34^ should be able to track more detailed changes in the electron-pair density as the molecule cascades quickly through the succession of states during the first 5 fs, followed by the slower build-up of final states at longer times. We anticipate that such measurements will not have to rely on the second-order interaction exploited here and will be carried out using a more traditional pump–probe set-up with two separate X-ray pulses^35–39^ once few- or sub-femtosecond pulses in the hard X-ray regime and equally short pump–probe delay times become routinely available at free-electron laser facilities. This will eventually permit the construction of a detailed temporal map of the changes in the radial electron-pair density by varying the delay time between the two pulses.

In summary, we report the first direct measurement of an ultrafast change in the radial electron-pair density of a molecule undergoing rapid dynamics, imaging the rearrangement of correlated electrons in response to an external perturbation. The observations are complementary to spectroscopy and allow characteristic length scales to be deduced. The measurement agrees well with detailed theoretical modelling. We anticipate that emerging technical improvements at free-electron laser facilities will enable the extraction of fully time-resolved real-space information in future experiments, either via temporal ghost-imaging analysis^40^ or by employing an X-ray pump X-ray probe set-up. Moreover, the use of hard X-rays for triggering dynamics, as demonstrated here, opens a path to the study of highly excited and otherwise inaccessible states of atoms and molecules and their dynamics. Following ionization by photons with energies that broadly range from the extreme ultraviolet to hard X-rays and beyond, Auger–Meitner decay is an important process responsible for radiation damage. Detailed real-space measurements on isolated molecules will improve mechanistic understanding. This may benefit areas such as radiobiology^41^, radiation therapy^42^ or single-particle imaging and serial femtosecond crystallography where radiation damage is responsible for the loss of contrast^43–50^. Overall, the current experiment points to new and exciting opportunities for ultrafast real-space imaging of rapid electronic transformations in matter, which play an important role across chemistry, biology and physics.

## Methods

To access the required average intensity regime of 10^17^–10^18^ W cm^−^^2^, the experiment was performed at the Coherent X-ray Imaging (CXI) end station of the LCLS. The temporal structures of the pulses were recorded with the X-band Transverse deflecting mode CAVity (XTCAV) at the CXI. The experiment employed X-ray pulses of, on average, 31.9 fs full-width at half-maximum duration and 9.486 keV mean photon energy. The focused X-ray beam was passed through a 2.4-mm-long sample cell that contained gaseous sulfur hexafluoride (SF_6_), and scattered photons were detected on a Jungfrau 4M X-ray detector situated approximately 8 cm downstream from the sample cell. The gas pressure was kept at 10.8 ± 0.2 torr. By analysing the scattering signal as a function of the recorded pulse energies, we found that focusing the X-ray beam to a diameter of approximately 2.14 μm yielded X-ray pulses in the desired intensity range. The experiment gathered data with 1.50 million X-ray pulses and, once all thresholding conditions were applied, 1.19 million shots were retained for further analysis. Pulses with low energies, as well as those with mean photon energies outside the narrow range of 9.477–9.496 keV, were discarded because chromatic aberration of the compound refractive lenses increased the X-ray beam diameter and thereby reduced the probability of sequential photoionization and scattering. Hence, pulses with energies between 0.22 mJ and 2.04 mJ were selected. This amounts to 171,757 out of the 1.19 million shots and corresponds to average intensities between 1.92 × 10^17^ W cm^−^^2^ and 1.78 × 10^18^ W cm^−^^2^. Within this range, only first- and second-order interactions between the X-ray photons and the target molecules were observed. Scattering signals were recorded within the momentum-transfer interval from 0.27 Å^−1^ to 4.27 Å^−1^, limited by the mean photon energy and the detector geometry. The data were binned in both the radial momentum-transfer coordinate and pulse energy. The experimental scattering signal and the corresponding hole in the radial electron-pair density were furthermore simulated with a kinetic model that approximates the dynamics of the Auger–Meitner decay, considers convolution with the average temporal pulse profile obtained with the XTCAV, and involves ab initio electronic structure calculations of state-specific molecular scattering probabilities. Further details are given in Supplementary Sections 4 and 5.

## Online content

Any methods, additional references, Nature Portfolio reporting summaries, source data, extended data, supplementary information, acknowledgements, peer review information; details of author contributions and competing interests; and statements of data and code availability are available at https://doi.org/10.1038/s41567-026-03363-8.

## Supplementary information

Supplementary Information Supplementary Discussion, Figs. 1–7 and Tables 1 and 2.

## Figures and Tables

**Extended Data Fig. 1:**
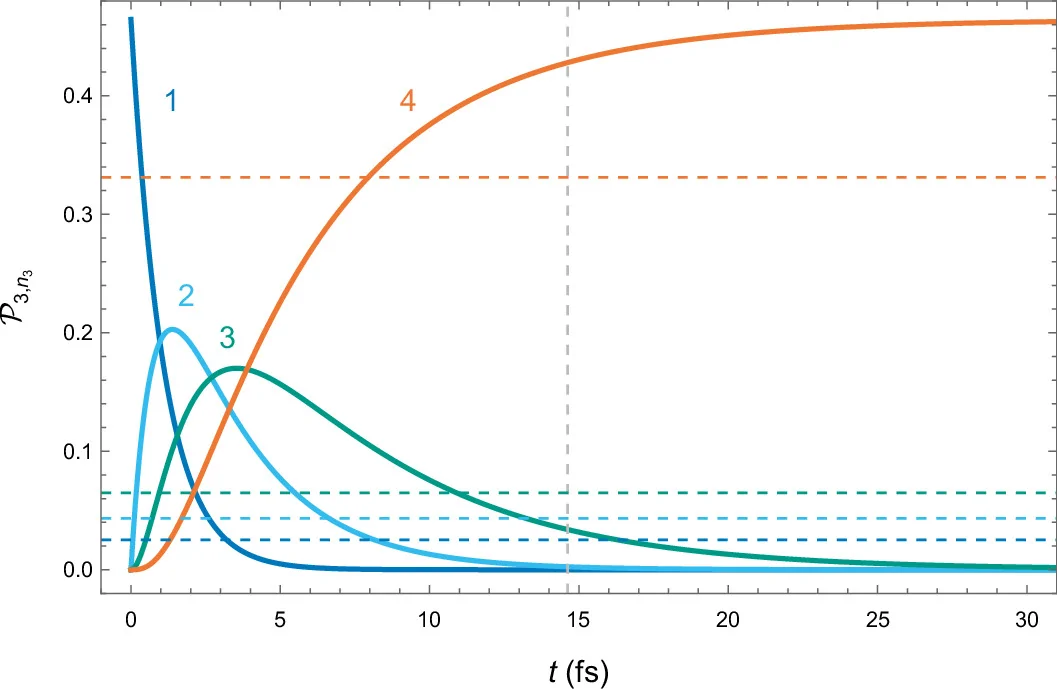
Modelled population dynamics of an Auger-Meitner decay channel of core-ionized SF_6_. The initial S(1s)^−1^ core vacancy (1) decays within 5 fs into a S(2p)^−2^ hole (2), which then decays further into a S(2p)^−1^ V^−2^ configuration (3) where V refers to valence orbitals. Finally, population in states with V^−4^ configuration (4) builds up. The average pump-probe delay time, 〈*δ*〉 ≈ 14.6 fs, is shown by a vertical dashed line. The effective populations given in Supplementary Table S1 are furthermore displayed by vertical dashed lines.

## Data Availability

The source data for Figs. 2 and 3 and Extended Data Figs. 1–3 are available via figshare at https://doi.org/10.6084/m9.figshare.32316573 (ref. ^51^). The repository also provides MOLPRO input and output files, as well as the calculated scattering signals of the individuals states that enter the kinetic model of the Auger–Meitner decay. Further data are available from the corresponding authors upon reasonable request.

## Code availability

The Wolfram Mathematica notebook used to prepare Figs. 2 and 3 and Extended Data Figs. 1–3 is available via figshare at https://doi.org/10.6084/m9.figshare.32316573 (ref. ^51^). Additional scripts for the processing and analysis of the experimental data are available from the corresponding authors upon reasonable request.
