# Supplementary material for: Real-space imaging of the electron-pair density hole in molecular Auger–Meitner decay
Source: Nat Phys. 2026 Jun 30;22(9):1473–8. doi: 10.1038/s41567-026-03363-8 (PMC13558058; doi:10.1038/s41567-026-03363-8)
Supplement: Supplementary file 1 [file 41567_2026_3363_MOESM1_ESM.pdf]

nature physics

Supplementary information

https://doi.org/10.1038/s41567-026-03363-8

Real-space imaging of the electron-pair density hole in molecular Auger–Meitner decay

In the format provided by the authors and unedited

Contents

S1 Absorption and Scattering Probabilities **1**
S1.1 Absorption Probability . . . 1
S1.2 Scattering Probability . . . 2

S2 Pulse Energy Determination and Diode Offset **3**
S2.1 Incorporation of the Diode Offset into the Analysis . . . 5
S2.2 Offset Determination . . . 5

S3 The Effective Beam Diameter **6**

S4 Density Distribution of Pump-Probe Delay Times **7**
S4.1 Idealized Gaussian Pulse . . . 7
S4.2 XTCAV Data . . . 7

S5 Model for Exponential Decay **8**
S5.1 Decay Channels, Lifetimes, Weights, and Effective Populations . . . 9
S5.2 Computational Details . . . 10
S5.3 Electronic States and Scattering Signals . . . 11
S5.4 Photo- and Auger-Meitner Electrons in Interaction Region . . . 14

S1 Absorption and Scattering Probabilities

The first two sections of the Supplementary Information discuss the absorption and scattering probabilities and show how the measured signal can be related to its underlying scattering probability.

S1.1 Absorption Probability

The transmitted intensity of X-ray that pass through a medium is given by an expression analogous to the well-known Beer-Lambert law, [1]

$$I \;=\; I_0 \, e^{-\mu T}, \tag{S1}$$

where I_0 is the incoming intensity of the X-ray beam, μ the linear attenuation coefficient, and T the length of the path the X-ray beam passes through medium. The linear attenuation coefficient can be calculated as

$$\mu \;=\; \rho \sum_i \, g_i \left(\frac{\mu}{\rho}\right)_i, \tag{S2}$$

where ρ is the density of the medium, g_i is the mass fraction of atom i, and $(\mu/\rho)_i$ is the mass absorption coefficient. The mass absorption coefficient, in turn, is given by

$$\left(\frac{\mu}{\rho}\right) \;=\; \frac{N_\mathrm{A}}{M}\,\sigma, \tag{S3}$$

with Avogadro's number N_A, the molecular weight M, and the total interaction cross section σ. These expressions define the total attenuation. If the total cross section is replaced with either an absorption or scattering cross section, one can calculate the light attenuation due to either of those processes instead. If the attenuation due to absorption is applied to an X-ray pulse passing through gas-phase molecules in a scattering cell, where T is equal to the length of the cell l, the exponent in Eq. (S1) becomes

$$-\mu T \;\approx\; -\rho \; l \sum_i \, \sigma_{\mathrm{a},i} \;=\; -\sigma_\mathrm{a}\;\rho\; l, \tag{S4}$$

where $\sigma_{\mathrm{a},i}$ is the absorption cross section of atom i and σ_a is the absorption cross section of the entire molecule. σ_a can be approximated by the sum of atomic absorption cross sections because photons in the hard X-ray regime predominantly ionize core electrons and the probability of such an ionization depends only weakly on an atom's chemical environment. Moreover, it is assumed here that the density ρ is homogeneous in the sample cell. Insertion of Eq. (S4) into Eq. (S1) now gives

$$\frac{I}{I_0} \;=\; e^{-\sigma_\mathrm{a}\rho l}. \tag{S5}$$

This exponential factor describes the probability of an X-ray photon passing through the sample cell without being absorbed. The probability of absorption is consequently unity minus the probability of transmission and we can write

$$n_\mathrm{a} \;=\; \left(1 \;-\; e^{-\sigma_\mathrm{a}\rho l}\right)\, n_0, \tag{S6}$$

where n_0 is the number of photons of the incident X-ray pulse and n_a is the number of absorbed photons. We note that attenuation due to absorption and scattering are small in the hard X-ray regime and typically amounts to less than 1%. Under these conditions, the Taylor expansion of the exponential function in Eq. (S6) can be truncated after the second term and we get

$$n_\mathrm{a} \;=\; \left(1 \;-\; 1 \;+\; \sigma_\mathrm{a}\;\rho\; l \;-\; \ldots\right)\, n_0 \;\approx\; \sigma_\mathrm{a}\;\rho\; l\; n_0. \tag{S7}$$

S1.2 Scattering Probability

Applying the same treatment and approximation as in Eq. (S7) to the scattering signal of the molecule in its electronic ground state, the coefficient $c_1(q)$, *i.e.* the linear contribution in Eq. (1) of the manuscript, can be written as,

$$c_1(q) \;=\; \rho\, l\, \left\langle \frac{d\sigma_\mathrm{s}}{d\Omega} \right\rangle_\phi . \tag{S8}$$

Here, $d\sigma_\mathrm{s}/d\Omega$ is the differential scattering cross section of the molecule in its electronic ground state and Ω is the solid angle into which an X-ray photon is scattered. The brackets around the cross section, $\langle \ldots \rangle_\phi$, imply averaging over the azimuthal detector angle ϕ. Note that, within the framework of the Waller-Hartree approximation and for a given mean-photon energy, the solid angle Ω into which a photon is scattered defines the magnitude and direction of the momentum transfer vector $\boldsymbol{q}$. While the directional information of $\boldsymbol{q}$ is partially lost due to azimuthal averaging, $\langle d\sigma_\mathrm{s}/d\Omega\rangle_\phi$ still depends on the vector magnitude $q = |\boldsymbol{q}|$ and thus responsible for the q-dependence of $c_1(q)$. For non-resonant scattering, this azimuthally averaged differential scattering cross section is, [2–4]

$$\left\langle \frac{d\sigma_\mathrm{s}}{d\Omega} \right\rangle_\phi = \; r_\mathrm{e}^2 \; \left\langle |\boldsymbol{\epsilon}_u \cdot \boldsymbol{\epsilon}_v^*|^2 \right\rangle_\phi \; S_0(q), \tag{S9}$$

where $|\boldsymbol{\epsilon}_u \cdot \boldsymbol{\epsilon}_v^*|^2$ is the X-ray polarization factor that is defined as the dot product of the polarization vectors of the incident and scattered photons, $\boldsymbol{\epsilon}_u$ and $\boldsymbol{\epsilon}_v$, $r_\mathrm{e} = e^2/(4\pi\varepsilon_0 m_\mathrm{e} c^2)$ is the classical electron radius, the product $r_\mathrm{e}^2\; |\boldsymbol{\epsilon}_u \cdot \boldsymbol{\epsilon}_v^*|^2$ is the differential Thomson scattering cross section, and $S(q)$ is the isotropic structure factor of the molecule in its electronic ground state. The polarization factor depends on the scattering angle θ_s and on the azimuthal detector angle ϕ relative to $\boldsymbol{\epsilon}_u$. For linearly polarized photons such as those generated at LCLS, the polarization factor is $|\boldsymbol{\epsilon}_u \cdot \boldsymbol{\epsilon}_v^*|^2 = \sin^2(\phi) + \cos^2(\phi)\cos^2(\theta_\mathrm{s})$ and $\langle |\boldsymbol{\epsilon}_u \cdot \boldsymbol{\epsilon}_v^*|^2\rangle_\phi = \big(1 + \cos^2(\theta_\mathrm{s})\big)/2$ after azimuthal averaging. [4–6] Eq. (S9) furthermore contains the isotropic molecular structure factor $S_0(q)$ of the molecule in its electronic ground state. The molecular structure factor $S_0(q)$ is often approximated by application of the independent atom model. [1] Here, we calculate it more accurately by means of *ab initio* molecular electronic structure theory *via* [4, 7, 8]

$$S_0(q) \;=\; N_\mathrm{e} \;+\; 2 \int_0^\infty \mathrm{sinc}(q r_{12})\; \Gamma_0(r_{12})\; dr_{12}. \tag{S10}$$

In Eq. (S10) above, N_e refers to the molecule's number of electrons and $\Gamma_0(r_{12})$ is the radial electron-pair density of the electronic ground state as a function of the electron-pair distance r_{12}. Section S5.2 provides further details on how $S_0(q)$ and its underlying electronic wavefunction are computed.

With Eqs. (S8) and (S9), the linear coefficient $c_1(q)$ becomes,

$$c_1(q) \;= r_\mathrm{e}^2\; \rho\; l\; \left\langle |\boldsymbol{\epsilon}_u \cdot \boldsymbol{\epsilon}_v^*|^2 \right\rangle_\phi \; S_0(q). \tag{S11}$$

Similarly, the quadratic coefficient in Eq. (1) of the manuscript, $c_2(q)$, is

$$c_2(q) \;=\; r_\mathrm{e}^2\; \rho\; l\; \frac{\sigma_\mathrm{a}}{2\pi w^2}\; \left\langle |\boldsymbol{\epsilon}_u \cdot \boldsymbol{\epsilon}_v^*|^2 \right\rangle_\phi \; \Big(S_+(q) - S_0(q)\Big), \tag{S12}$$

with the absorption cross section σ_a and the beam waist of the X-ray pulse w. Moreover, $S_+(q)$ is the effective molecular structure factor of the Auger-Meitner decaying ion and defined by Eq. (S10) if the electron-pair density of the neutral molecule, $\Gamma_0(r_{12})$, is replaced with the effective electron-pair

density of the ion, $\Gamma_+(r_{12})$. Further details on the calculation of $S_+(q)$ and all its components are provided again in Section S5.2. We finally note that spatial focusing of the X-ray pulse implies a decrease of w and therefore leads to an increase of $c_2(q)$.

Eq. (S11) gives the scattering probability $P_\mathrm{s}(q)$ at a given value of momentum transfer q. In an experiment, however, the scattering probability is usually binned and averaged within a range of radial q-values, *i.e.*, within concentric rings of finite width on the detector. Such a binning implies integration of the scattering probability in Eq. (S11) on the interval $[q_1, q_2]$ where q_1 and q_2 refer to the lower and upper limits of the binning in q, [9]

$$P_\mathrm{s}(q_1, q_2) \;\propto\; r_\mathrm{e}^2\, \rho\, l\, \frac{2\pi}{k_0^2} \int_{q_1}^{q_2} q\, \left\langle |\boldsymbol{\epsilon}_u \cdot \boldsymbol{\epsilon}_v^*|^2 \right\rangle_\phi\, S(q)\, dq. \tag{S13}$$

Eq. S13 provides a dimensionless value proportional to the probability of scattering between q_1 and q_2. The azimuthally averaged experimental scattering signal is typically expressed in photons per pixel or photons per steradian. The following considerations allow us to express Eq. (S13) as a probability per steradian. The integral in the unnormalized expression in Eq. (S13) involves area element $dA = 2\pi/k_0^2\, q\, dq$ which, when integrated from q_1 to q_2, gives the normalization factor $\pi/k_0^2\,(q_2^2 - q_1^2)$. Multiplication of the right-hand side of Eq. (S13) with this factor leads to the scattering probability per pixel. The scattering patterns are recorded on a flat detector perpendicular to the propagation of the X-rays. Both the distance to the q bin from the interaction region and the effective area of the pixel that detects the scattered photons vary. Pixels near the edge of the detector have a smaller effective area and larger distance from the interaction region. To account for this, a geometry factor of $\cos^3(\theta_\mathrm{s})$ is applied. [5] Additionally, data is typically processed into quantities per pixel. A conversion factor of z^2/A_px for pixel area A_px and interaction region to detector distance z in addition to this geometry correction converts these per pixel quantities into quantities per steradian. With all these considerations in mind, the isotropic molecular structure factor $S(q)$ can be related to what is measured on the detector. The probability of scattering to a pixel between q_1 and q_2 is given by,

$$P_\mathrm{s}^\mathrm{px}(q_1, q_2) \;=\; r_\mathrm{e}^2\, \rho\, l\, \frac{2A_\mathrm{px}}{z^2(q_2^2 - q_1^2)} \int_{q_1}^{q_2} q\, \left\langle |\boldsymbol{\epsilon}_u \cdot \boldsymbol{\epsilon}_v^*|^2 \right\rangle_\phi\, \cos^3(\theta_\mathrm{s})\, S(q)\, dq. \tag{S14}$$

S2 Pulse Energy Determination and Diode Offset

The pulse energy is determined by correlating the signals from a gas detector that measures the pulse energy and a more precise photodiode upstream of the interaction region in diagnostics section 2. [10] With this correlation, the photodiode reading can be converted into a pulse energy. Moreover, the mean-photon energy E_ph is determined with a detector that interacts with the electron beam after it emitted the X-ray pulse. With the pulse energy and mean-photon energy, the number of photons n_0 can be calculated shot-by-shot.

The gas detector, which measures the pulse energy, contains nitrogen which ionizes and fluoresces as the X-ray pulse passes through. Two photomultiplier tubes measure and amplify the fluoresced photons. A voltage is normally applied to the photomultiplier tubes such that the signal does not saturate. In this experiment, these voltages were not perfectly set, which led to saturated signal at higher pulse energies (Fig. S1b). The low pulse energy values prior to saturation were used to convert the photodiode readings to pulse energy (Fig. S1c).

There is a small energy offset ΔE in the photodiode that arises from imperfect zeroing which leads to more complicated expressions for the measured signal. The relationships between the true pulse

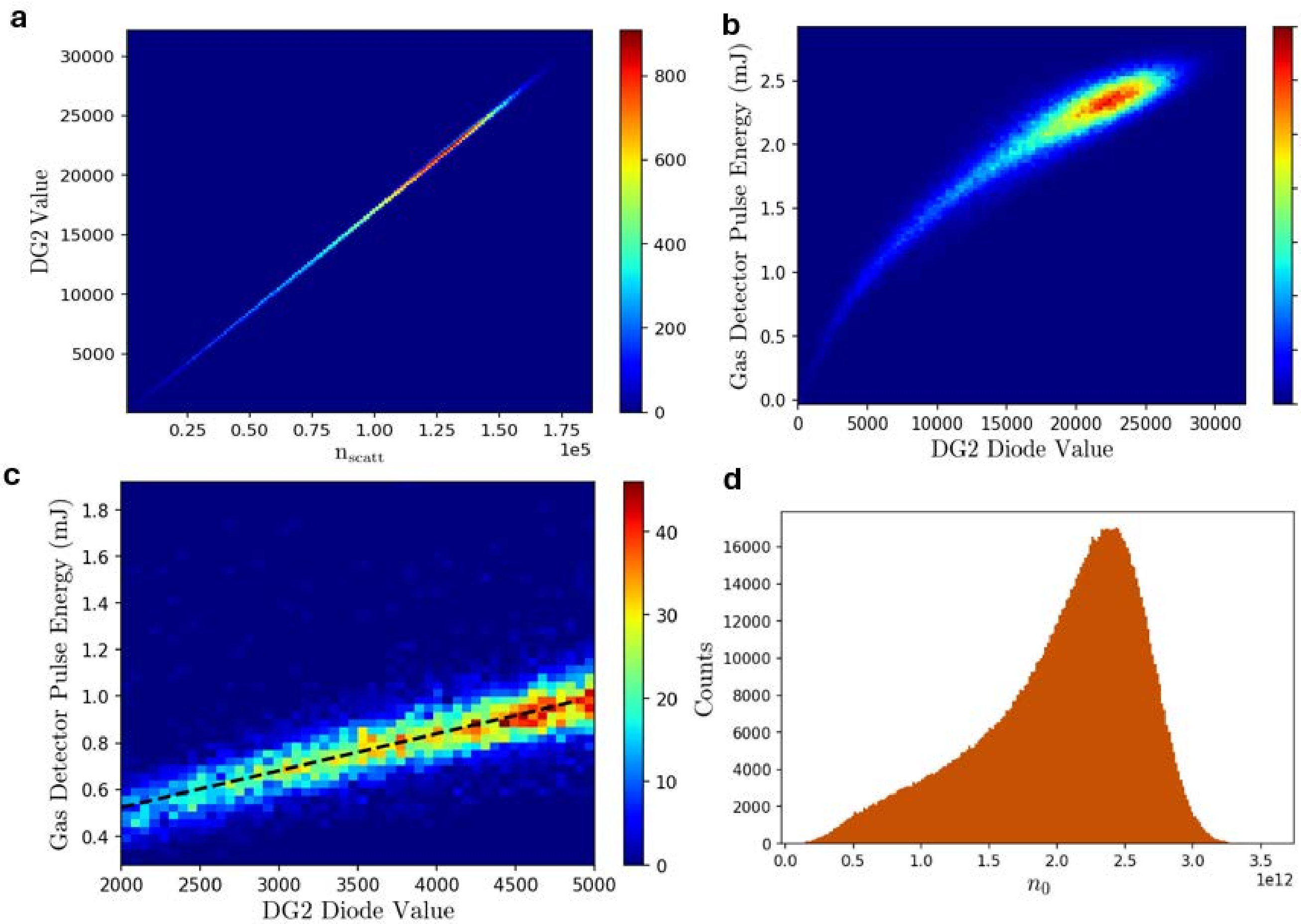

Figure S1: The pulse energy is calculated using the correlation between a precise upstream photodiode and a gas detector measuring the pulse energy. The photodiode is labelled 'DG2' in the plots in reference to the section of CXI where the photodiode is located. **(a)** The correlation between the photodiode reading and the integrated scattering signal as measured by the Jungfrau detector is shown for 100 000 X-ray shots. The strong linear correlation reflects the reliability of the photodiode measurement. **(b)** The correlation between the pulse energy measured with the detector and the photodiode is shown for 100 000 shots. While the correlation is linear at low pulse energy, the detector saturates at higher pulse energy and the linear correlation is lost. **(c)** The same correlation is shown at low pulse energy values before the onset of saturation of the gas detector. These low pulse energy readings are used to convert the photodiode values to pulse energy. **(d)** The final distribution of calculated n_0 values is shown for all 1 190 000 shots retained after thresholding conditions.

energy E_0 and the measured pulse energy E_D as well as the photon number equivalents n_0, n_D, and Δn are given by,

$$E_0 = E_\mathrm{D} + \Delta E, \tag{S15}$$

$$n_0 = n_\mathrm{D} + \Delta n = \frac{E_\mathrm{D}}{E_\mathrm{ph}} + \frac{\Delta E}{E_\mathrm{ph}}. \tag{S16}$$

S2.1 Incorporation of the Diode Offset into the Analysis

The presence of the diode offset affects the analysis *via* Eq. (1) in the manuscript. With the diode offset, the number of photons scattered onto the detector is given as,

$$n_J(q) \;=\; D(q) \;+\; \rho l \left\langle \frac{d\sigma_{\mathrm{s}}}{d\Omega} \right\rangle_{\phi} (n_{\mathrm{D}} + \Delta n) \;+\; \frac{\sigma_{\mathrm{a}}\rho l}{2\pi w^2} \left(\left\langle \frac{d\sigma_{\mathrm{s}}^{+}}{d\Omega} \right\rangle_{\phi} - \left\langle \frac{d\sigma_{\mathrm{s}}}{d\Omega} \right\rangle_{\phi} \right) (n_{\mathrm{D}} + \Delta n)^2, \quad \text{(S17)}$$

where $D(q)$ is the background scattering on the Jungfrau detector and $d\sigma_{\mathrm{s}}^{+}/d\Omega$ is the differential scattering cross section of the photoionized molecule that is undergoing Auger-Meitner decay. The coefficients of the quadratic fit, $n_{\mathrm{s}}(q) \approx c_0(q) + c_1(q) n_{\mathrm{D}} + c_2(q) n_{\mathrm{D}}^2$, are now,

$$c_0(q) \;=\; D(q) \;+\; \rho l \left\langle \frac{d\sigma_{\mathrm{s}}}{d\Omega} \right\rangle_{\phi} \Delta n \;+\; \frac{\sigma_{\mathrm{a}}\rho l}{2\pi w^2} \left(\left\langle \frac{d\sigma_{\mathrm{s}}^{+}}{d\Omega} \right\rangle_{\phi} - \left\langle \frac{d\sigma_{\mathrm{s}}}{d\Omega} \right\rangle_{\phi} \right) \Delta n^2, \quad \text{(S18)}$$

$$c_1(q) \;=\; \rho l \left\langle \frac{d\sigma_{\mathrm{s}}}{d\Omega} \right\rangle_{\phi} \;+\; \frac{\sigma_{\mathrm{a}}\rho l}{\pi w^2} \left(\left\langle \frac{d\sigma_{\mathrm{s}}^{+}}{d\Omega} \right\rangle_{\phi} - \left\langle \frac{d\sigma_{\mathrm{s}}}{d\Omega} \right\rangle_{\phi} \right) \Delta n, \quad \text{(S19)}$$

$$c_2(q) \;=\; \frac{\sigma_{\mathrm{a}}\rho l}{2\pi w^2} \left(\left\langle \frac{d\sigma_{\mathrm{s}}^{+}}{d\Omega} \right\rangle_{\phi} - \left\langle \frac{d\sigma_{\mathrm{s}}}{d\Omega} \right\rangle_{\phi} \right). \quad \text{(S20)}$$

S2.2 Offset Determination

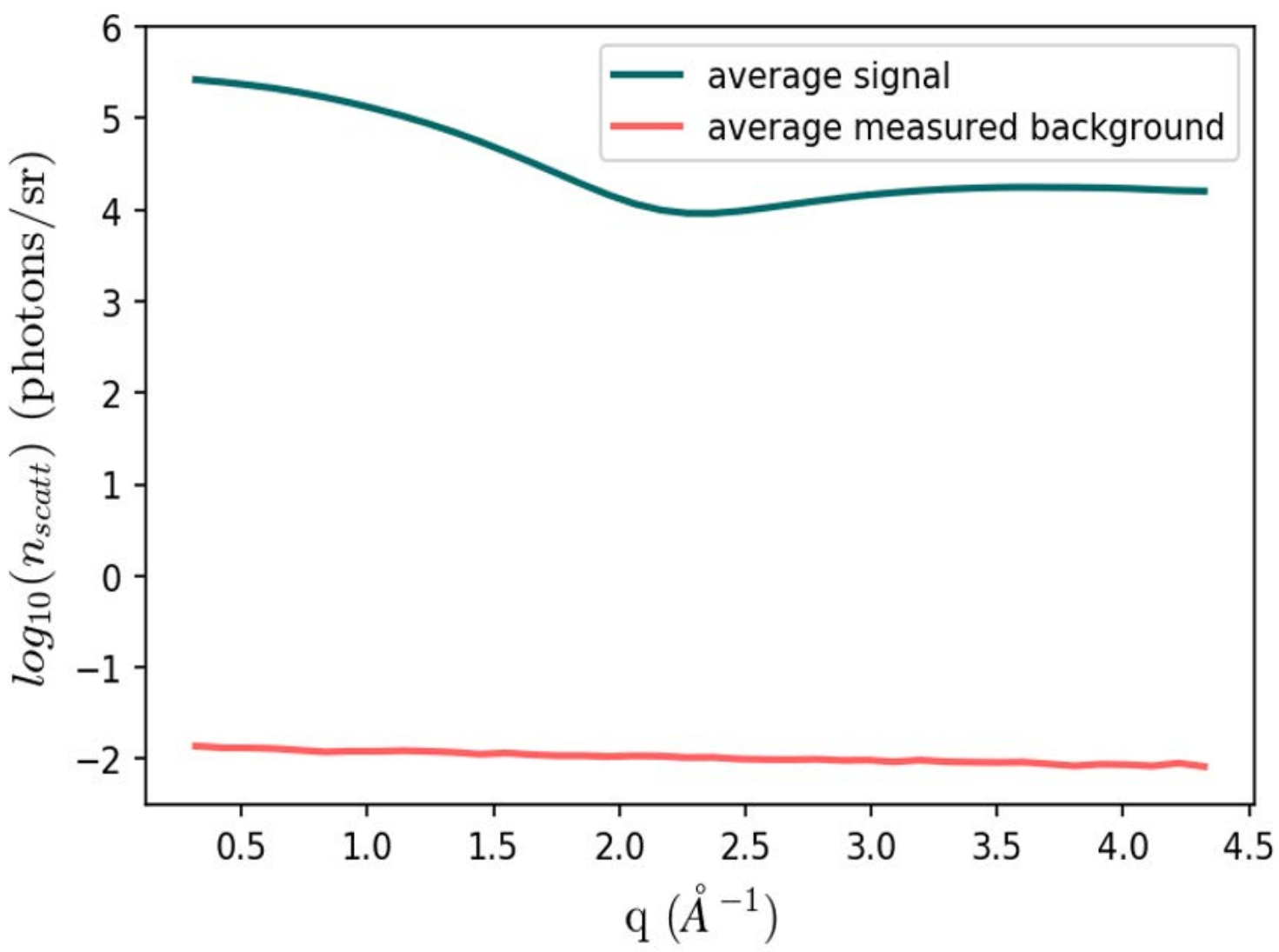

Figure S2: Logarithm of the average scattering signal of SF_6 for all ~ 1.5 million measured shots and for a 7 399 shot background run in which no gas was in the sample cell but scattering off the experimental apparatus was measured. The background counts constitute a very small portion of the signal, typically about 6 orders of magnitude less than the scattering signal.

If expressed in terms of the other coefficients, $c_0(q)$ takes the form,

$$c_0(q) \;=\; D(q) \;+\; c_1(q)\,\Delta n \;-\; c_2(q)\,\Delta n^2\,. \quad \text{(S21)}$$

Fig. S2 shows that the background counts $D(q)$ are exceptionally low. Therefore, while Δn is small, the contributions of Δn and Δn^2 are the largest contributors to $c_0(q)$. The offsets ΔE and Δn can

be well approximated by minimizing the following expression with Δn as the variable,

$$\sum_q \left| c_0(q) \; - \; \big(c_1(q)\; \Delta n \; - \; c_2(q)\; \Delta n^2\big)\right|. \tag{S22}$$

Rather than integrating continuously over q, the sum in Eq. (S22) considers the discrete q bins because the coefficients $c_0(q)$, $c_1(q)$, and $c_2(q)$ are extracted from q-binned data. It is assumed here that Eq. (S22) converges to the background counts when minimized. These counts are very low.

S3 The Effective Beam Diameter

At intensities where photoabsorption followed by scattering becomes non-negligible, the respective signal of subsequent absorption and scattering can be treated similarly to two-photon absorption. [11] The spatial and temporal profiles of the X-ray pulse are approximated by Gaussians, with standard deviations w and τ for the spatial and temporal beam profiles. The densities of the neutral and photoionized molecule are functions of space and time. Initially, all molecules are in the electronic ground state and, as the pulse passes through, the molecules are partially photoionized, the fraction depending on the local fluence of the pulse. The number of photoionized molecules produced, or ground state molecules depleted, is equal to the number of photons absorbed from the X-ray pulse. If the fraction of X-ray photons absorbed and the fraction of ground state molecules depleted is small, the scattering signal that probes the photoionized system can be approximated as the quadratic function shown in Eq. (1) in the manuscript. If the photoionization fraction is too large, the quadratic approximation breaks down and the scattering formula becomes,

$$\begin{aligned} n_{\mathrm{s}}(q) \;=\; & \left\langle \frac{d\sigma_{\mathrm{s}}^{+}}{d\Omega} \right\rangle_{\phi} \rho l \; n_0 \;+\; 2\pi \left(\left\langle \frac{d\sigma_{\mathrm{s}}}{d\Omega} \right\rangle_{\phi} - \left\langle \frac{d\sigma_{\mathrm{s}}^{+}}{d\Omega} \right\rangle_{\phi} \right) \rho l \; \frac{I_{\mathrm{peak}}}{E_{\mathrm{ph}}} \\ & \times \int_0^{\infty} \exp\!\left(-\frac{\sqrt{\pi}}{2}\; \tau \; \sigma_{\mathrm{a}} \; e^{-\frac{2r^2}{w^2}} \; \frac{I_{\mathrm{peak}}}{E_{\mathrm{ph}}} \right) e^{-\frac{2r^2}{w^2}} \; r \; dr \\ & \times \int_{-\infty}^{\infty} \exp\!\left(-\frac{\sqrt{\pi}}{2}\; \tau \; \sigma_{\mathrm{a}} \; e^{-\frac{2r^2}{w^2}} \; \mathrm{erf}\!\left(\frac{t}{\tau}\right) \frac{I_{\mathrm{peak}}}{E_{\mathrm{ph}}} \right) e^{-\frac{t^2}{\tau^2}} \; dt, \end{aligned} \tag{S23}$$

where the peak intensity is given by $I_{\mathrm{peak}} = 2E_{\mathrm{ph}}/(\pi w^2 \sqrt{\pi}\tau)\, n_0$.

To compare the experimental and computational results, the parameter w was optimized to match the predicted signal Eq. (S23) to the measured signal. A bootstrapping analysis was applied, in which the error bars of the experimental signal were used to generate 1000 signal that were fitted to Eq. (S23), yielding an array of 1000 optimized w values. The mean and standard deviation of this array were taken to be the optimal w value and its error. The w value thus determined was (0.968 ± 0.014) μm, which corresponds to an effective beam diameter of 2.28 μm *via* the relationship $\mathrm{FWHM} = 2\sqrt{2\ln 2}w$. Alternatively, the parameter w can be determined by Eq. (2) in the manuscript. Using Eq. (2), the parameter w was optimized such that the mean absolute deviation between the measured and simulated percent difference scattering signals became minimal. The value of w determined in this manner was found to be 0.909 μm, which corresponds to an effective beam diameter of $\varpi \approx 2.14$ μm. The values of the beam diameter determined in these two different ways are thus remarkably consistent.

S4 Density Distribution of Pump-Probe Delay Times

Given the intensity-time profile of the incident X-ray pulse, $I(t)$, the probability density distribution of pump-probe delay times between two photons can be obtained by

$$\varrho(\delta) \;=\; \frac{1}{N_\varrho} \int_{-\infty}^{\infty} I\Big(\tau - \frac{\delta}{2}\Big)\; I\Big(\tau + \frac{\delta}{2}\Big)\; d\tau, \tag{S24}$$

where $\tau = (t_1 + t_2)/2$ is the mean time of two photons at times $t_1 = \tau - \delta/2$ and $t_2 = \tau + \delta/2$ and $\delta = t_2 - t_1$ is the delay time, *i.e.* the time that passes between the first and second photon hitting the target. Moreover, the factor N_ϱ ensures normalization so that $\varrho(\delta)$ can be interpreted as a probability density. It is given as

$$N_\varrho \;=\; \int_{0}^{\infty} \int_{-\infty}^{\infty} I\Big(\tau - \frac{\delta}{2}\Big)\; I\Big(\tau + \frac{\delta}{2}\Big)\; d\tau\; d\delta. \tag{S25}$$

With the probability density distribution of pump-probe delay times in Eq. (S24), the average pump-probe delay time of the pulse is

$$\big\langle \delta \big\rangle \;=\; \int_{0}^{\infty} \delta\; \varrho(\delta)\; d\delta. \tag{S26}$$

S4.1 Idealized Gaussian Pulse

For an idealized pulse with Gaussian-shaped envelope,

$$I_{\mathcal{G}}(t) \;=\; \frac{2}{\eta} \sqrt{\frac{\ln 2}{\pi}}\; e^{-4\ln 2\; \frac{(t-\xi)^2}{\eta^2}}, \tag{S27}$$

centred at time ξ and with full-width-at-half-maximum duration η, analytic expressions can be derived for $\varrho(\delta)$ and $\langle\delta\rangle$. Inserting Eq. (S27) into Eq. (S24) and solving the integral yields,

$$\varrho_{\mathcal{G}}(\delta) \;=\; \frac{1}{\eta} \sqrt{\frac{\ln 2}{2\pi}}\; 4^{1-\frac{\delta^2}{\eta^2}}. \tag{S28}$$

Insertion of Eq. (S28) into Eq. (S26) now leads to an average pump-probe delay time of

$$\big\langle \delta \big\rangle_{\mathcal{G}} \;=\; \frac{\eta}{\sqrt{\pi \ln 4}} \;\approx\; 0.479\; \eta. \tag{S29}$$

S4.2 XTCAV Data

For a real intensity-time profile $I(t)$ measured with the XTCAV and averaged over many shots, $\varrho(\delta)$ and $\langle\delta\rangle$ can be calculated numerically. The respective functions $I(t)$ and $\varrho(\delta)$ are shown in Extended Data Figure 2.

S5 Model for Exponential Decay

Assuming an exponential decay of a core-ionized state *via* $\mathcal{N}_{\rm ch}$ dominating channels with $\mathcal{N}_a$ steps of an Auger-Meitner cascade per channel, the time-dependent population of a particular state at a given step n within channel a can be modelled as

$$\mathcal{P}_{a,n_a}(t) \;=\; \begin{cases} \mathcal{W}_a\; e^{-\Gamma_{a,n_a} t/\hbar}, & \text{if } n_a = 1, \\ \mathcal{W}_a\; e^{-\Gamma_{a,n_a} t/\hbar}\; \prod_{i_a=1}^{n_a-1} \left(1 - e^{-\Gamma_{a,i_a} t/\hbar}\right), & \text{if } 1 < n_a < \mathcal{N}_a, \\ \mathcal{W}_a\; \prod_{i_a=1}^{\mathcal{N}_a} \left(1 - e^{-\Gamma_{a,i_a} t/\hbar}\right), & \text{if } n_a = \mathcal{N}_a. \end{cases} \tag{S30}$$

Here, $\mathcal{W}_a$ is the weight of channel a. The sum of all weights for all channels equals unity,

$$\sum_{a}^{\mathcal{N}_{\rm ch}} \mathcal{W}_a \;=\; 1. \tag{S31}$$

Moreover, $\Gamma_{a,n_a}/\hbar$ is the inverse lifetime of the state at step n_a within channel a. With the probability density distribution of pump-prone delay times $\varrho(\delta)$ defined in Eq. (S24), the effective population of the state the X-ray pulse prepares and probes can be obtained by weighting $\mathcal{P}_{a,n_a}(\delta)$ with $\varrho(\delta)$,

$$\left\langle \mathcal{P}_{a,n_a} \right\rangle \;=\; \int_0^\infty \mathcal{P}_{a,n_a}(\delta)\; \varrho(\delta)\; d\delta. \tag{S32}$$

With the effective populations $\left\langle \mathcal{P}_{a,n_a} \right\rangle$ at hand, the relative difference scattering signal can now be estimated as

$$\frac{\Delta S(q)}{S(q)} \;=\; \sum_{a=1}^{\mathcal{N}_{\rm ch}} \sum_{n_a=1}^{\mathcal{N}_a} \left\langle \mathcal{P}_{a,n_a} \right\rangle \frac{S_{a,n_a}(q) - S_0(q)}{S_0(q)}. \tag{S33}$$

Here, the first sum runs over all $\mathcal{N}_{\rm ch}$ decay channels and the second sum runs over all $\mathcal{N}_a$ steps of the respective channel. $S_{a,n_a}(q)$ is the isotropic total scattering signal of the state at step n_a within channel a at radial momentum transfer coordinate q. $S_0(q)$ is the scattering signal of the neutral molecule in its electronic ground state and equilibrium geometry prior to interaction with the pulse.

The model in Eq. (S33) relies on three fundamental assumptions: first, the decay is exponential and can be modelled with Eq. (S30). Second, the scattering signal at each step in a given channel is well represented by a single electronic state, *i.e.* branching of the channels does not have to be considered explicitly. Third, nuclear dynamics can be considered frozen. The first assumption is usually well justified for Auger-Meitner decay. Because the populations in Eq. (S32) are averaged over the probability density of delay times rather than being considered at absolute delay times, a single exponential function is sufficient to describe the decay of each state. [12] The second assumption is justified if the scattering signals of different electronic states that contribute at a given step are similar. Consequently, the scattering signal of the chosen state should be at least qualitatively similar to the weighted average of all relevant states. The discussion in Section S5.3 demonstrates that this condition is fulfilled almost exactly for all core-excited states with the same amount of vacancies in the same shell, even across different decay channels, and still holds well for states with the same amount of vacancies in the valence. Finally, the third assumption is justified as long as the effective pump-probe delay time of the X-ray pulse in Eq. (S5.3) is shorter than the timescale of the nuclear motion. Here, we have an average delay time of $\langle \delta \rangle \approx 14.6$ fs. Given that nuclear dynamics of second and third row elements usually proceeds on timescales of 10–100 fs [13] and that strongly dissociative states with vacancies in bonding orbitals ($5a_{1g}$, $4t_{1u}$, $5t_{1u}$) are populated only after some additional delay, it is thus unlikely that nuclear dynamics contributes significantly to the measured scattering signal. To a good approximation, the third assumption is thus justified as well.

S5.1 Decay Channels, Lifetimes, Weights, and Effective Populations

Considering the sulfur *KLL* Auger-Meitner spectrum of SF_6, [14, 15] we assume that the decay of the hole in the S(1s) orbital is dominated by the following three channels, [16]

$$\begin{aligned}
&\mathrm{S(1s)}^{-1} \xrightarrow{\Gamma_{1,1}} \mathrm{S(2s)}^{-2} \xrightarrow{\Gamma_{1,2}} \mathrm{S(2s)}^{-1}\,\mathrm{S(2p)}^{-1}\,\mathrm{V}^{-1} \xrightarrow{\Gamma_{1,3}} \mathrm{S(2p)}^{-2}\,\mathrm{V}^{-2} \xrightarrow{\Gamma_{1,4}} \mathrm{S(2p)}^{-1}\,\mathrm{V}^{-4} \xrightarrow{\Gamma_{1,5}} \mathrm{V}^{-6},\\
&\mathrm{S(1s)}^{-1} \xrightarrow{\Gamma_{2,1}} \mathrm{S(2s)}^{-1}\,\mathrm{S(2p)}^{-1} \xrightarrow{\Gamma_{2,2}} \mathrm{S(2p)}^{-2}\,\mathrm{V}^{-1} \xrightarrow{\Gamma_{2,3}} \mathrm{S(2p)}^{-1}\,\mathrm{V}^{-3} \xrightarrow{\Gamma_{2,4}} \mathrm{V}^{-5},\\
&\mathrm{S(1s)}^{-1} \xrightarrow{\Gamma_{3,1}} \mathrm{S(2p)}^{-2} \xrightarrow{\Gamma_{3,2}} \mathrm{S(2p)}^{-1}\,\mathrm{V}^{-2} \xrightarrow{\Gamma_{3,3}} \mathrm{V}^{-4},
\end{aligned}$$

where the exponents denote the number of vacancies in the S(1s), S(2s), S(2p), and valence orbitals. Due to its weak intensity and similarity to the last channel after its second step, we disregard the direct decay of the $\mathrm{S(1s)}^{-1}$ hole into the valence *via* the $\mathrm{S(2s)}^{-1}\,\mathrm{V}^{-1}$ configuration.

Similarly, the fluorine *KLL* Auger-Meitner spectra of LiF, NaF, and MgF_2 [17] suggest that the decay of the hole in the F(1s) orbital is dominated by the following three channels,

$$\begin{aligned}
&\mathrm{F(1s)}^{-1} \xrightarrow{\Gamma_{4,1}} \mathrm{F(2s)}^{-2},\\
&\mathrm{F(1s)}^{-1} \xrightarrow{\Gamma_{5,1}} \mathrm{F(2s)}^{-1}\,\mathrm{V}^{-1},\\
&\mathrm{F(1s)}^{-1} \xrightarrow{\Gamma_{6,1}} \mathrm{V}^{-2}.
\end{aligned}$$

Drawing from an analogy to Ne and F^-, further decay of the $\mathrm{F(2s)}^{-1}$ hole into the valence is assumed to be energetically forbidden. This assumption is also justified by electronic structure calculations: the energies of electronic states with $\mathrm{F(2s)}^{-1}\,\mathrm{V}^{-2}$ and V^{-3} configurations are usually higher than the energies of the $\mathrm{F(2s)}^{-2}$ and $\mathrm{F(2s)}^{-1}\,\mathrm{V}^{-1}$ configurations, respectively. We note, however, that the steps $\mathrm{F(2s)}^{-2} \to \mathrm{F(2s)}^{-1}\,\mathrm{V}^{-2}$ and $\mathrm{F(2s)}^{-1}\,\mathrm{V}^{-1} \to \mathrm{V}^{-3}$ are just slightly above the threshold and thus cannot be ruled out with certainty. Considering the small contribution of these channels to the measured scattering signal and the low kinetic energy of the Auger-Meitner electrons potentially ejected in these steps, further decay should not notably affect the scattering signal.

The weights $\mathcal{W}_a$ of the six decay channels, the lifetimes $\hbar/\Gamma_{a,n_a}$ of the hole states, and the effective populations $\langle \mathcal{P}_{a,n_a} \rangle$ used in the model are summarized in Tab. S1. The time-dependent populations of the third decay channel, $\mathcal{P}_{a,n_a}(t)$, and the respective effective populations, $\langle \mathcal{P}_{3,n_3} \rangle$, are displayed in Extended Data Figure 1.

The total lifetime of the $\mathrm{S(1s)}^{-1}$ hole state of 1.1 fs given in Tab. S1 is taken from the experimentally measured line width of the photoelectron ejected from the S(1s) orbital of H_2S, $\Gamma \approx 0.6$ eV. [14] Similarly, the total lifetime of the $\mathrm{F(1s)}^{-1}$ hole state of 3.4 fs is deduced from the scaled line width of the F(1s) photoelectron of HF, $\Gamma \approx 0.194$ eV, calculated by Schimmelpfennig, Nestmann, and Peyerimhoff. [19] All other lifetimes of the $\mathrm{S(1s)}^{-1}$ decay channels are taken directly from Grånäs *et al.* who have inferred them from the more extensively studied Auger-Meitner decay of Argon. [16]

**hole after photoionization**	$\mathbf{S(1s)^{-1}}$			$\mathbf{F(1s)^{-1}}$		
**photoionization cross section**	3.05×10^{-21} cm^2			0.29×10^{-21} cm^2		
**relative photoionization**	63.6%			36.4%		
**relative intensities**	4.5%	22.5%	73.0%	5.6%	22.2%	72.2%
**Weights of Channels**	$\mathcal{W}_1$	$\mathcal{W}_2$	$\mathcal{W}_3$	$\mathcal{W}_4$	$\mathcal{W}_5$	$\mathcal{W}_6$
	2.9%	14.3%	46.4%	2.0%	8.1%	26.2%
**Approximate Lifetimes**	$\hbar/\Gamma_{1,n_1}$	$\hbar/\Gamma_{2,n_2}$	$\hbar/\Gamma_{3,n_3}$	$\hbar/\Gamma_{4,n_4}$	$\hbar/\Gamma_{5,n_5}$	$\hbar/\Gamma_{6,n_6}$
step: $n_a = 1$	1.1 fs	1.1 fs	1.1 fs	3.4 fs	3.4 fs	3.4 fs
step: $n_a = 2$	0.3 fs	0.3 fs	2.8 fs			
step: $n_a = 3$	0.3 fs	2.8 fs	5.6 fs			
step: $n_a = 4$	2.8 fs	5.6 fs				
step: $n_a = 5$	5.6 fs					
**Effective Populations**	$\langle \mathcal{P}_{1,n_1} \rangle$	$\langle \mathcal{P}_{2,n_2} \rangle$	$\langle \mathcal{P}_{3,n_3} \rangle$	$\langle \mathcal{P}_{4,n_4} \rangle$	$\langle \mathcal{P}_{5,n_5} \rangle$	$\langle \mathcal{P}_{6,n_6} \rangle$
step: $n_a = 1$	0.155%	0.775%	2.513%	0.323%	1.275%	4.139%
step: $n_a = 2$	0.009%	0.046%	4.331%	1.724%	6.809%	22.108%
step: $n_a = 3$	0.007%	1.297%	6.493%			
step: $n_a = 4$	0.254%	1.994%	33.108%			
step: $n_a = 5$	0.398%	10.204%				
step: $n_a = 6$	2.041%					

Table S1: Weights, lifetimes, and effective populations assumed in the model of the Auger-Meitner decay of photoionized SF_6. The weights of the six channels are estimated from the photoionization cross sections of sulfur and fluorine [18], interpolated to the mean-photon energy of the X-ray pulse of 9.486 keV, as well as from the line intensities in the *KLL* Auger-Meitner spectra of sulfur in SF_6 [14] and fluorine in Mg_2 [17]. The approximate lifetimes of the holes are taken from Refs. [14] and [16] for sulfur and from Ref. [19] for fluorine. The effective populations are estimated from the weights, lifetimes, and the XTCAV data shown in Extended Data Figure 2 according to Eq. (S32).

S5.2 Computational Details

All electronic structure calculations are performed with MOLPRO 2015.1. [20, 21] The equilibrium geometry of SF_6 in its electronic ground state is optimized with CCSD(T)/aug-cc-pV5Z-DK [22–26] in D_{2h} symmetry, employing a 10th order Douglas-Kroll-Hess (DKH) Hamiltonian with exponential parametrization to account for scalar-relativistic effects [27]. This geometry is used throughout.

The electronic energies and wavefunctions of the core- and valence-hole states are calculated at an effective Hartree-Fock level, using the multi-configurational self-consistent field (MCSCF) program MULTI of MOLPRO [28, 29], again employing the DKH Hamiltonian and an aug-cc-pVTZ-DK basis set. In each case, the orbital occupancy of the state is specified explicitly via the *occ*, *closed*, and *restrict* cards of the MULTI program. The active space is restricted to approximate each state by a linear combination of configuration state functions that only differ in their distribution of spin over the open-shell orbitals while conserving the total spin multiplicity of the wavefunction. In many cases, only a single configuration state function was created. To avoid variational collapse into energetically lower-lying eigenstates of the respective ion, several calculations are run to iteratively

optimize the orbitals. [30] In the first step, the hole orbitals are frozen and only the fully occupied orbitals are optimized. In the second step, only the hole orbitals are optimized while all other occupied orbitals that transform as the same irreducible representations as the hole orbitals are kept frozen. This procedure is repeated once or twice until convergence of the eigenenergy was achieved. Each step used the optimized orbitals of the previous step as a starting point. All calculations of states with vacancies in either the sulfur core or the valence are run in D_{2h} symmetry. Calculations of pure fluorine core-hole states are run without symmetry and the canonical molecular orbitals of the neutral molecule are localized according to the Boys criteria prior to the MCSCF-based calculation of the core-hole state. The wavefunction of the neutral molecule was calculated at the Hartree-Fock level of theory. All orbital coefficients are stored in a molden file for later use.

The total isotropic scattering signals are calculated from the electronic eigenstates $\left|\Psi_{a,n_a}\right\rangle$ as, [2, 4]

$$S_{a,n_a}(q) \;=\; N_\mathrm{e} \;+\; \left\langle \Psi_{a,n_a} \middle| \hat{\mathcal{L}}(q) \middle| \Psi_{a,n_a} \right\rangle, \tag{S34}$$

where N_e refers to the number of electrons of the molecule in the respective state, the braket implies integration over all electronic coordinates, and $\hat{\mathcal{L}}(q)$ is the isotropic two-electron scattering operator,

$$\hat{\mathcal{L}}(q) \;=\; \sum_{n}^{N_\mathrm{e}} \sum_{m \neq n}^{N_\mathrm{e}} \operatorname{sinc}\big(q \cdot (r_n - r_m)\big). \tag{S35}$$

Here, q is the norm of the momentum-transfer vector, r_m and r_n are the radial coordinates of the electrons with indices m and n, and the sums run over all N_e electrons. The matrix element in Eq. (S34) is evaluated analytically with an in-house code developed in Oxford. [3, 31–33]

S5.3 Electronic States and Scattering Signals

Figs. S3–S7 show percent difference scattering signals of different electronic states or channels involved in the model of the Auger-Meitner decay of SF_6 relative to the scattering signal of the neutral molecule in its electronic ground state.

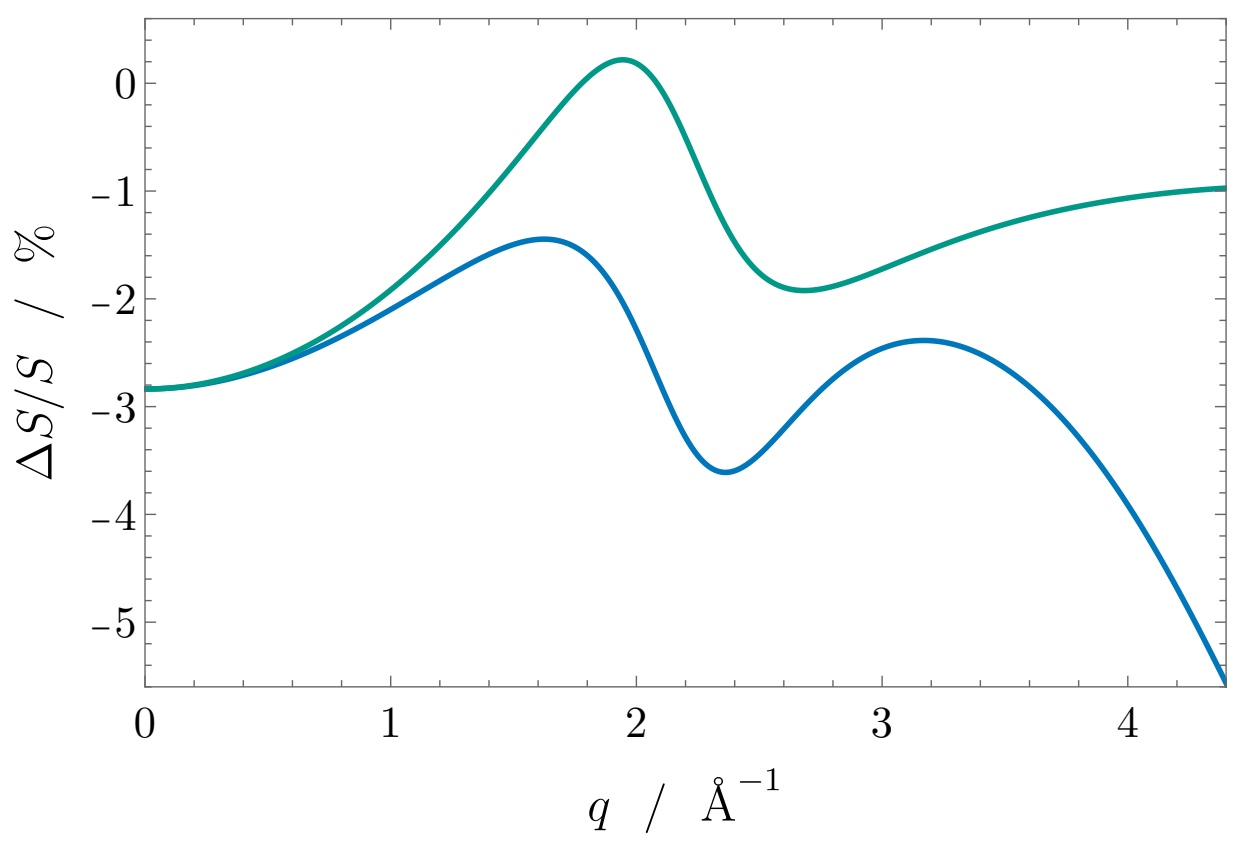

Figure S3: Percent difference X-ray scattering signals, $\Delta S/S$, of the $S(1s)^{-1}$ and $F(1s)^{-1}$ core hole states of SF_6.

Fig. S3 displays the signals of the initial $S(1s)^{-1}$ and $F(1s)^{-1}$ hole states. While both scattering curves clearly reflect the loss of one electron with roughly −2.8% at $q = 0$ Å^{-1} and have similar shapes, the difference signal of the $S(1s)^{-1}$ hole is generally stronger and drops significantly at large

values of momentum transfer. This conforms with the fact that an S(1s) electron is more compact and stronger bound than an F(1s) electron and that more electrons adjust to the removal of an S(1s) electron in the sulfur core than to the removal of an F(1s) electron in the fluorine core.

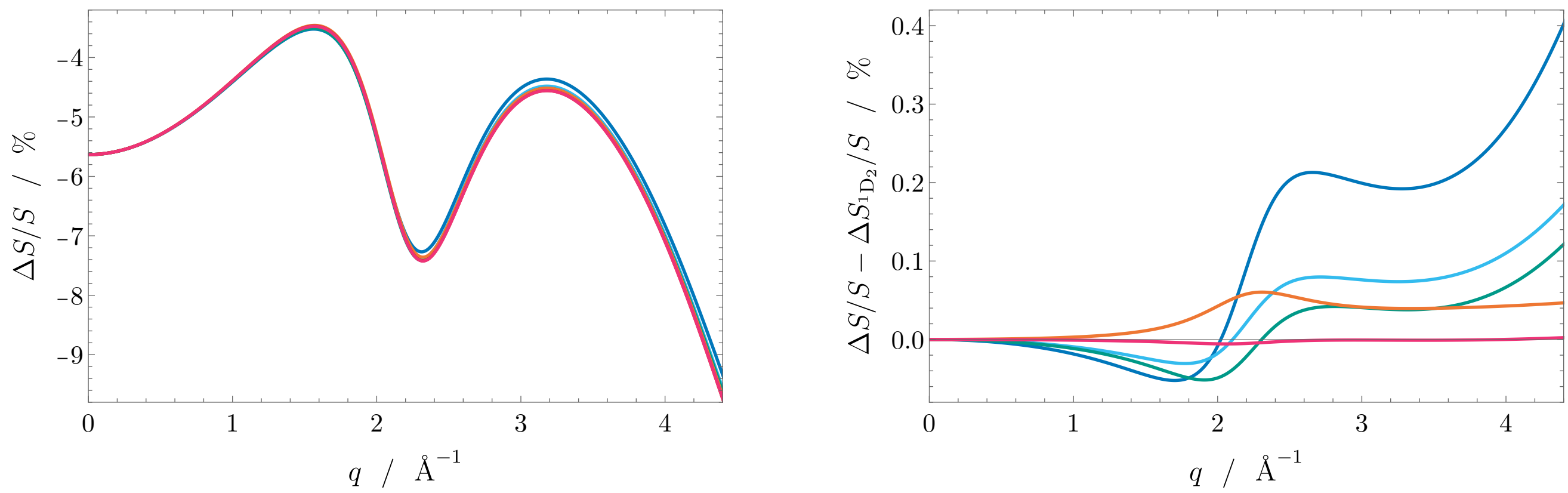

Figure S4: **(Left)** Percent difference X-ray scattering signals, $\Delta S/S$, of different states of SF_6 with a double vacancy in the sulfur L shell. **(Right)** Differences of the same differential X-ray scattering signals and the signal of the $^1D_2$ state. **(Left and Right)** $^1S_0$, $^1P_1$, ^3P, $^1S_0$, $^1D_2$, ^3P.

Fig. S4 displays the signals of six different states with double vacancies in the sulfur L shell that get populated in the first steps of the three channels of the Auger-Meitner decay of the $S(1s)^{-1}$ hole. The curves are almost indistinguishable, which implies that, in principle, any of the states could be chosen to represent the scattering of the $S(2s)^{-2}$, $S(2s)^{-1}$ $S(2p)^{-1}$, and $S(2p)^{-2}$ configurations. Given that the lines of the $^1P_1$ and $^1D_2$ states are the most intense in the KLL Auger-Meitner spectrum of SF_6, we have chosen those states to represent the respective $S(2s)^{-1}$ $S(2p)^{-1}$ and $S(2p)^{-2}$ configurations in our model.

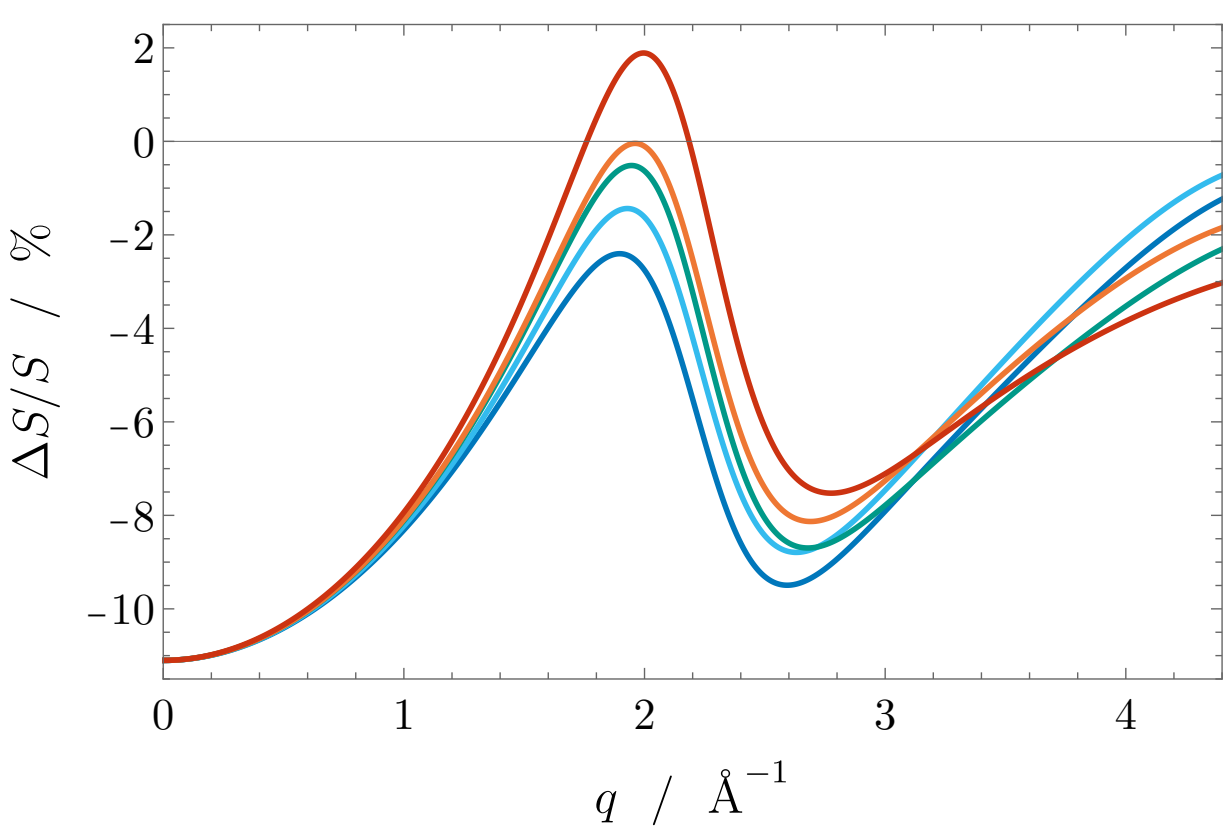

Figure S5: Percent difference X-ray scattering signals, $\Delta S/S$, of different singlet states of SF_6 with a quadruple vacancy in valence orbitals with significant amplitude on the sulfur atom. The hole configurations of the respective singlet states are $(5a_{1g})^{-2}(4t_{1u})^{-2}$, $(4t_{1u})^{-4}$, $(5a_{1g})^{-2}(5t_{1u})^{-2}$, $(4t_{1u})^{-2}(5t_{1u})^{-2}$, and $(5t_{1u})^{-4}$.

Fig. S5 shows the signals of five different singlet states with quadruple vacancies in the valence that can get populated in the last step of the third channel of the Auger-Meitner decay of the $S(1s)^{-1}$ hole. All chosen vacancies reside in bonding orbitals with significant amplitude on the sulfur atom. The

scattering curves are rather similar but consistently show a shift of the local maximum at around $q \approx 2$ Å^{-1} to larger values if the vacancies are moved from stronger to weaker bound orbitals. Similarly, albeit not as consistently, the signals are shifted towards lower values at larger values of q. Given that the $4t_{1u}$ orbital is triply degenerate, whereas the $5a_{1g}$ is not, and that it has stronger amplitude on the sulfur atom than the $5t_{1u}$ orbital, and furthermore considering that the $4t_{1u}$ lies energetically between the the $5a_{1g}$ and $5t_{1u}$ orbitals, we have chosen this orbital to represent the valence vacancies in all three decay channels of the $S(1s)^{-1}$ hole decay. Fig. S5 further suggests that this is a reasonable choice. The scattering signal of the state with the $(4t_{1u})^{-4}$ configuration lies somewhat in between the curves of the states with $(5a_{1g})^{-2}(4t_{1u})^{-2}$, $(5a_{1g})^{-2}(5t_{1u})^{-2}$, and $(4t_{1u})^{-2}(5t_{1u})^{-2}$ configurations, while the state with the $(5t_{1u})^{-4}$ configuration differs more strongly, having the largest local maximum around at around $q \approx 2$ Å^{-1}. That said, all five scattering curves are qualitatively very similar. The choice of a particular configuration to represent the scattering signal of a state with vacancies in the valence orbitals thus leads to some uncertainty in the quantitative scattering signal but has little consequence for its qualitative shape.

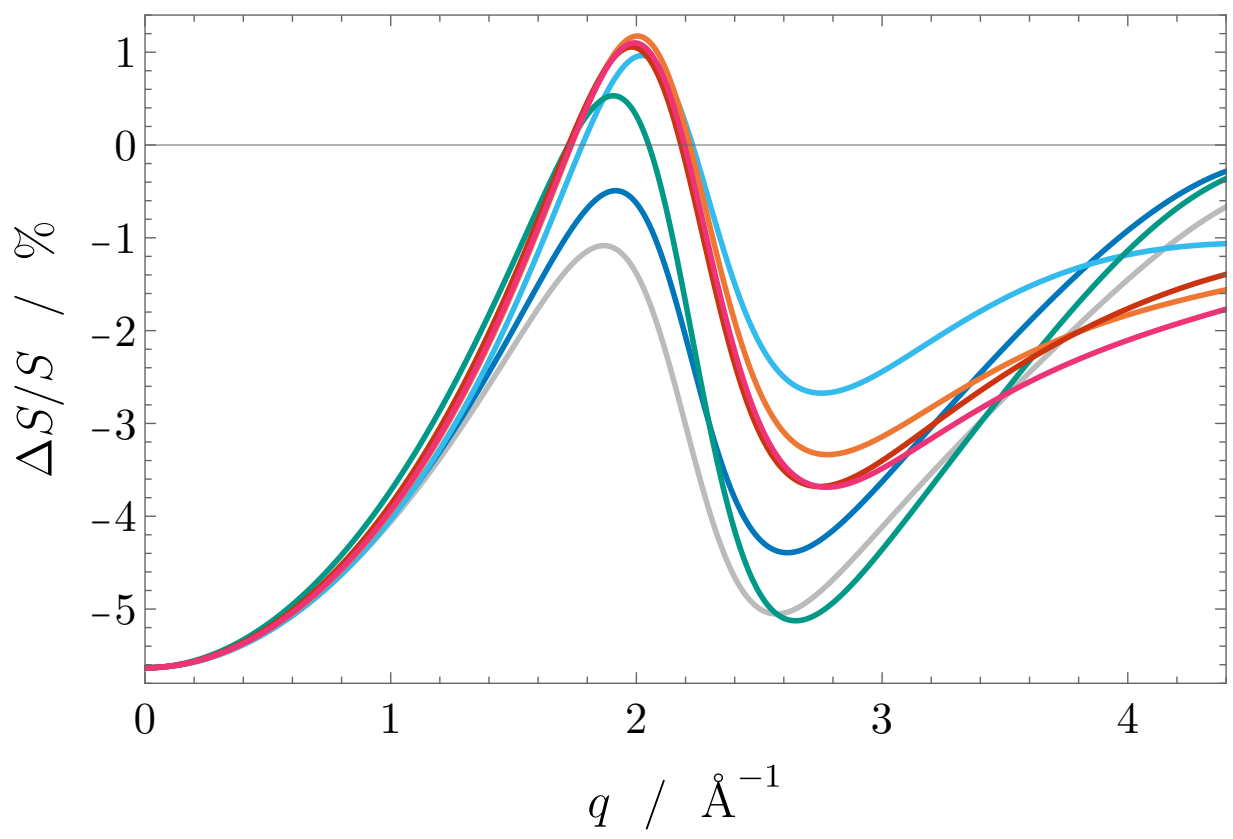

Figure S6: Percent difference X-ray scattering signals, $\Delta S/S$, of different singlet states of SF_6 with a double vacancy in valence orbitals with significant amplitude on the fluorine atom. The hole configurations of the respective singlet states are $(5a_{1g})^{-2}$, $(4t_{1u})^{-2}$, $(1t_{2g})^{-2}$, $(3e_g)^{-2}$, $(1t_{2u})^{-2}$, $(5t_{1u})^{-2}$, and $(1t_{1g})^{-2}$.

Similar to Fig. S5, Fig. S6 shows the signals of seven different singlet states with double vacancies in the valence that can get populated in the third channel of the Auger-Meitner decay of the $F(1s)^{-1}$ hole. Here, the chosen valence orbitals include all bonding as well as non-bonding orbitals with significant amplitude on the fluorine atom. As in Fig. S5 before, the scattering curves are quite similar but show a shift of the local maximum at around $q \approx 2$ Å^{-1} to larger values and tend to drop to lower values at larger values of q if the vacancies are moved from stronger to weaker bound orbitals. The signals of the states with $(1t_{2u})^{-2}$, $(5t_{1u})^{-2}$, and $(1t_{1g})^{-2}$ hole configurations are almost indistinguishable and the signals of the states with $(5a_{1g})^{-2}$ and $(4t_{1u})^{-2}$ configurations are most different. Given that and the fact that the $1t_{2u}$ orbital is triply degenerate, has particularly strong amplitude on the fluorine atom, and lies energetically in between the $1t_{2g}$, $3e_g$, $5t_{1u}$, and $1t_{1g}$ orbitals, we have chosen this orbital to represent the valence vacancies in the two respective channels of the $F(1s)^{-1}$ hole decay. Again, all curves are qualitatively very similar and the particular choice of valence orbitals has little impact on the overall shape of the scattering signal.

Finally, Fig. S7 shows the percent difference scattering signals of all six decay channels. The signals are averaged over all steps of a given channel. To allow a direct comparison of the curves, the

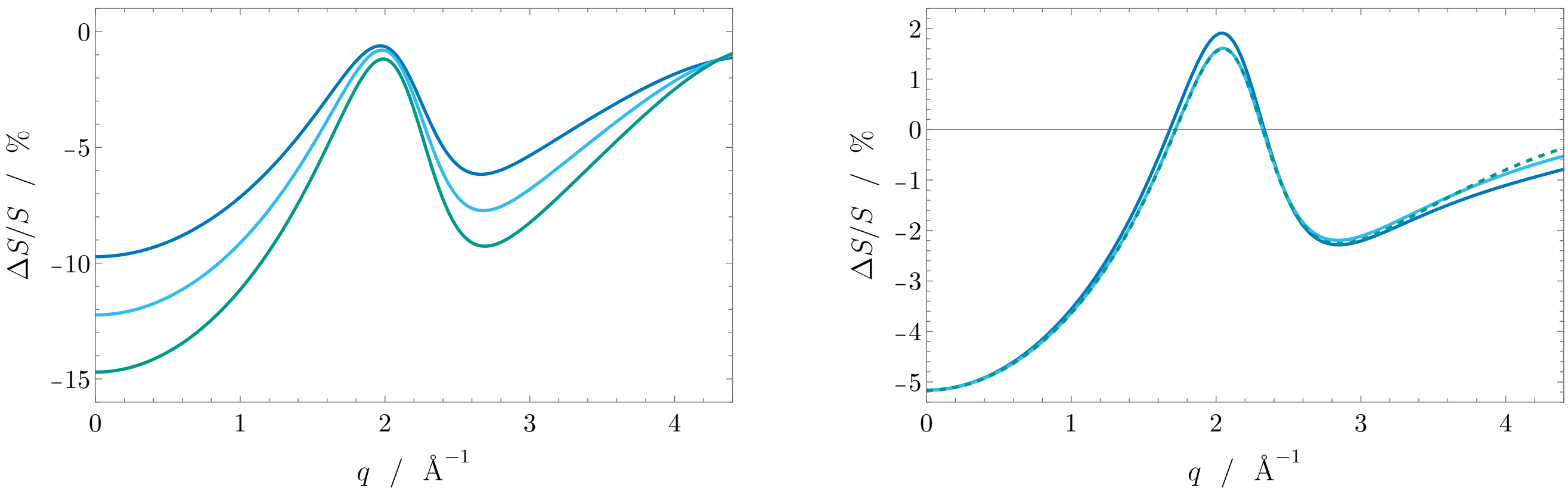

Figure S7: **(Left)** Percent difference X-ray scattering signals, $\Delta S/S$, of the three different channels for the decay of the $S(1s)^{-1}$ hole of SF_6, modelled with valence vacancies in the $4t_{1u}$ orbital. The channels decay via the $S(2p)^{-2}$, $S(2s)^{-1}S(2p)^{-1}$, and $S(2s)^{-2}$ hole configurations. **(Right)** Differential X-ray scattering signals, $\Delta S/S$, of the three different channels for the decay of the $F(1s)^{-1}$ hole of SF_6, modelled with valence vacancies in the $1t_{2u}$ orbital. The channels decay into the V^{-2}, $F(2s)^{-1}V^{-1}$, and $F(2s)^{-2}$ hole configurations. **(Left and Right)** For the sake of comparison, the effective populations are independently normalized for each channel, *i.e.* every channels is effectively weighted by unity. The scattering signals also include the incoherent contribution from the photoelectrons and Auger-Meitner electrons that remain in the interaction region until the second X-ray photon is scattered.

effective populations $\langle \mathcal{P}_{a,n_a} \rangle$ are normalized for each channel such that the signals are all weighted by unity rather than $\mathcal{W}_a$. The scattering signals include the incoherent contribution of the ejected electrons that remain in the interaction region. The scattering signals of the three channels of the $S(1s)^{-1}$ hole decay (left) are very similar and differ mainly in their magnitudes. The first channel that decays via the $S(2s)^{-2}$ hole configuration displays to the strongest percent difference scattering signal, which reflects that this channel leads to the build-up of up to six vacancies in the valence. The third channel that decays via the $S(2p)^{-2}$ hole configuration, in contrast, only leads to the build-up of up to four vacancies and its scattering signal is consequently the weakest. (Note that the third channel contributes most strongly to the overall signal, given its large weight of $\mathcal{W}_3 \approx 46.4\%$ that is not included in Fig. S7.) The qualitative similarity of the scattering curves of all three channels furthermore reflects that the model is not particularly sensitive to the precise value of the weights, lifetimes, and effective populations. They mainly affect the overall magnitude of the combined difference signal but have only limited impact upon its shape. The same holds for the scattering signals of the three channels of the $F(1s)^{-1}$ hole decay (right). Since our model assumes that all three channels do not lead to the build-up of more than a double vacancy due to energy constraints, their respective percent difference scattering signals are effectively identical.

S5.4 Photo- and Auger-Meitner Electrons in Interaction Region

The kinetic energy of the ejected electrons, T_{a,n_a}, can be obtained from the total electronic energies of the calculated eigenstates, E_{a,n_a}. The kinetic energy of an Auger-Meitner electron is simply given as the difference in energy between two consecutive steps,

$$T_{a,n_a} = E_{a,n_a-1} - E_{a,n_a}, \tag{S36}$$

for $n_a > 1$. Similarly, the kinetic energy of the initial photoelectron is given by the difference between the mean-photon energy of the X-ray pulse, E_{ph}, and the electronic energy of the respective core-hole

state, $E_{a,1}$, the latter defined relative to the energy of the ground state of the neutral molecule,

$$T_{a,1} \;=\; E_{\rm ph} - E_{a,1}. \tag{S37}$$

The electronic energies of the eigenstates that represent the steps in the model of the Auger-Meitner cascade as well as the kinetic energies of the ejected electrons are summarized in Tab. S2. With the kinetic energies T_{a,n_a} at hand, the velocity of the ejected electrons, v_{a,n_a}, can be calculated as,

$$v_{a,n_a} \;=\; c\,\sqrt{1-\left(\frac{E_0}{E_0+T_{a,n_a}}\right)^2}, \tag{S38}$$

where c is the speed of light and E_0 the electron's rest energy,

$$E_0 \;=\; m_{\rm e}\,c^2, \tag{S39}$$

which relates to the electron's rest mass $m_{\rm e}$. Now, we can use the velocity v_{a,n_a} to transform the probability density distribution of pump-probe delay times $\varrho(\delta)$ in Eq. (S24) into a probability distribution of distances an ejected electron travels between the incidence of the two photons, $\rho_{a,n_a}\big(r/v_{a,n_a}\big)$. Here, $r/v_{a,n_a}$ refers to the time that passes when an electron with velocity v_{a,n_a} travels a distance r. For the photoelectron with $n_a = 1$, the probability distribution of travelled distances is simply,

$$\rho_{a,1}\big(r\big) \;=\; \frac{1}{2\;v_{a,1}}\;\varrho\big(r/v_{a,1}\big), \tag{S40}$$

where the prefactor ensures normalization of $\rho_{a,1}\big(r/v_{a,1}\big)$ over the full range of negative and positive distances. For the Auger-Meitner electrons with $n_a > 1$, the probability distribution of travelled distances can be defined as,

$$\rho_{a,n_a}\big(r\big) \;=\; \frac{1}{N_\rho}\int_0^\infty \mathcal{P}_{a,n_a}\big(t\big)\;\varrho\big(t + r/v_{a,n_a}\big)\;dt, \tag{S41}$$

where $\mathcal{P}_{a,n_a}\big(t\big)$ is the time-dependent population defined in Eq. (S30). It accounts for the fact that the Auger-Meitner electron is ejected with a probability equal to $\mathcal{P}_{a,n_a}\big(t\big)$ at time t after the X-ray photon was absorbed. The density $\varrho\big(t + r/v_{a,n_a}\big)$ is shifted accordingly, reflecting that the Auger-Meitner electron has, on average, less time to travel than the photoelectron before the second photon is scattered. The factor $1/N_\rho$ furthermore ensures normalization of $\rho_{a,n_a}\big(r\big)$ again,

$$N_\rho \;=\; 2\iint_0^\infty \mathcal{P}_{a,n_a}\big(t\big)\;\varrho\big(t + r/v_{a,n_a}\big)\;dt\;dr. \tag{S42}$$

The fraction of free electrons that remain in the interaction region can now be estimated as,

$$f_{a,n_a} \;=\; \frac{1}{N_{\mathcal{F}}}\int_0^\infty \mathcal{F}\big(r'\big)\int_{-\infty}^\infty \mathcal{F}\big(r\big)\;\rho_{a,n_a}\big(|r'-r|\big)\;dr\;dr'. \tag{S43}$$

Here, $\mathcal{F}\big(r'\big)$ describes the focus profile of the X-ray beam with Gaussian shape,

$$\mathcal{F}\big(r\big) \;=\; \frac{2}{\varpi}\sqrt{\frac{\ln 2}{\pi}}\;e^{-4\ln 2\;\frac{r^2}{\varpi^2}}, \tag{S44}$$

and FWHM beam diameter ϖ. The factor $1/N_{\mathcal{F}}$ in Eq. (S43) once more ensures normalization such that the fraction f_{a,n_a} will equal unity if the electron remains at the position at which it is ejected,

$$N_{\mathcal{F}} \;=\; \int_0^\infty \mathcal{F}\big(r'\big) \int_{-\infty}^\infty \mathcal{F}\big(r\big)\; \delta\big(r'-r\big)\; dr\; dr' \;=\; \int_0^\infty \mathcal{F}^2\big(r'\big)\; dr' \;=\; \sqrt{\frac{\ln 2}{2\pi}}\;\frac{1}{\varpi}. \tag{S45}$$

Here, $\delta\big(r'-r\big)$ is the Dirac delta function. The function $\mathcal{F}\big(r\big)$ in Eq. (S43) refers to the first photon that is absorbed at position r and the function $\mathcal{F}\big(r'\big)$ to the second photon that is scattered at position r' by an electron that travelled from r to r'.

The velocities of the photo- and Auger-Meitner electrons v_{a,n_a} and the respective fractions f_{a,n_a} calculated with the XTCAV data are given in Tab. S2. The fractions f_{a,n_a} are above 0.78 for the photoelectrons and almost unity for all Auger-Meitner electrons, taking values of at least 0.93 throughout. It can thus be assumed that most of the ejected electrons are still in the interaction region when the second X-ray photon is scattered. The scattering of the free electrons must therefore be taken into account. Their velocities are, however, sufficiently large that the scattering of the outgoing electron and of the parent cation becomes incoherent very quickly. Even the slowest electrons should travel around 20 nm within the 14.6 fs average delay time of the pulse. Hence, the isotropic total scattering signal of step n_a within channel a can be estimated as an incoherent sum of the scattering signal of the cation, $S^{\mathrm{Ion}}_{a,n_a}(q)$, and the total fraction of the ejected electrons that remain in the interaction region,

$$S_{a,n_a}(q) \;\approx\; S^{\mathrm{Ion}}_{a,n_a}(q) \;+\; \sum_{i_a}^{n_a} f_{a,i_a}. \tag{S46}$$

We note that the expression in Eq. (S43) is based upon the assumption that all ejected electrons travel orthogonal to the wave vector of the incident X-ray beam. While this is a reasonable assumption for the photoelectrons, it isn't necessarily true for the Auger-Meitner electrons, which may display a different angular distribution that depends on the symmetry of the particular transition as well as on the deflection of the electron by residual interaction with the final cation. [34] If the sample cell is longer then the beam diameter and if the attenuation of the X-ray beam along the sample cell is weak, the fraction in Eq. (S43) constitutes a lower limit for the number of electrons that remain in the interaction region. Here, the sample cell is 2.4 mm long, compared to a FWMH beam diameter of only $\varpi \approx 2.14$ μm. Moreover, the intensity of the X-ray beam barely changes along the sample cell, since both the photoabsorption and scattering cross sections are weak and the density of molecules is low. The fraction of the Auger-Meitner electrons that remain in the interaction region is thus likely to be larger than f_{a,n_a} in Eq. (S43). That said, f_{a,n_a} is already close to unity for all Auger-Meitner electrons (*see Tab. S2*) and the deviation of the real fractions of free electrons that remain in the interaction region from f_{a,n_a} must therefore be negligible. Under the conditions of the current experiment, Eqs. (S43) and (S46) are sufficiently accurate.

We add that, if the Gaussian delay density $\varrho_{\mathcal{G}}(\delta)$ defined in Eq. (S28) is employed, a simple analytical expression can be derived for the fraction of scattering photoelectrons,

$$f_{\mathcal{G},a,1} \;=\; \frac{1}{N_{\mathcal{F}}} \int_0^\infty \mathcal{F}(r') \int_{-\infty}^\infty \mathcal{F}(r)\; \varrho_{\mathcal{G},a,1}(r'-r)\; dr\; dr' \;=\; \frac{\varpi}{\sqrt{\varpi^2+v_{a,1}^2\eta^2}}. \tag{S47}$$

The fraction $f_{\mathcal{G},a,1}$ depends on the FWHM beam diameter ϖ, on the photoelectron's velocity $v_{a,1}$, and on the FWHM pulse duration η. If the beam diameter is much larger than the product of the

velocity and pulse duration, $\varpi \gg v_{a,1}\eta$, the fraction $f_{\mathcal{G},a,1}$ is close to unity. If $\varpi \ll v_{a,1}\eta$ instead, $f_{\mathcal{G},a,1}$ tends to zero. If we introduce the ratio $\zeta = \eta/\varpi$, Eq. (S47) further simplifies to,

$$f_{\mathcal{G},a,1} \;=\; \left(1 + \zeta^2 v_{a,1}^2\right)^{-1/2}. \tag{S48}$$

Here, we have $\zeta \approx 14.21$ fs/μm. Inserting that value and the two velocities $v_{a,1}$ from Tab. S2 into Eq. (S48), we obtain $f_{\mathcal{G},a,1} \approx 0.820$ and $f_{\mathcal{G},a,1} \approx 0.788$ for the S(1s) and F(1s) electrons, respectively. These values are very close to the fractions $f_{a,1}$ in Tab. S2. Eq. (S48) thus permits a quick back-of-the-envelope estimate of $f_{a,1}$. Remarkably, even the fractions of the free Auger-Meitner electrons with $n_a > 1$ can be approximated quite well with Eq. (S48). This can be attributed to the fact that all fractions are close to unity. The additional time it takes for the Auger-Meitner electron to be released after the first X-ray photon got absorbed has little effect, since the ratio of the pulse duration and beam diameter is already small compared to the velocities of the electrons.

**Eigenenergies**	E_{1,n_1}	E_{2,n_2}	E_{3,n_3}	E_{4,n_4}	E_{5,n_5}	E_{6,n_6}
step: $n_a = 1$	2501.5 eV	2501.5 eV	2501.5 eV	695.2 eV	695.2 eV	695.2 eV
step: $n_a = 2$	528.6 eV	475.9 eV	403.3 eV	97.0 eV	73.3 eV	45.0 eV
step: $n_a = 3$	497.0 eV	439.5 eV	255.4 eV			
step: $n_a = 4$	490.1 eV	303.6 eV	142.5 eV			
step: $n_a = 5$	361.0 eV	198.0 eV				
step: $n_a = 6$	261.6 eV					
**Kinetic Energies**	T_{1,n_1}	T_{2,n_2}	T_{3,n_3}	T_{4,n_4}	T_{5,n_5}	T_{6,n_6}
step: $n_a = 1$	6984.9 eV	6984.9 eV	6984.9 eV	8791.2 eV	8791.2 eV	8791.2 eV
step: $n_a = 2$	1972.9 eV	2025.6 eV	2098.1 eV	598.2 eV	621.9 eV	650.2 eV
step: $n_a = 3$	31.6 eV	36.4 eV	147.9 eV			
step: $n_a = 4$	6.9 eV	135.9 eV	112.9 eV			
step: $n_a = 5$	129.1 eV	105.7 eV				
step: $n_a = 6$	99.4 eV					
**Velocities**	v_{1,n_1}	v_{2,n_2}	v_{3,n_3}	v_{4,n_4}	v_{5,n_5}	v_{6,n_6}
step: $n_a = 1$	$49.07~\frac{\text{nm}}{\text{fs}}$	$49.07~\frac{\text{nm}}{\text{fs}}$	$49.07~\frac{\text{nm}}{\text{fs}}$	$54.90~\frac{\text{nm}}{\text{fs}}$	$54.90~\frac{\text{nm}}{\text{fs}}$	$54.90~\frac{\text{nm}}{\text{fs}}$
step: $n_a = 2$	$26.27~\frac{\text{nm}}{\text{fs}}$	$26.61~\frac{\text{nm}}{\text{fs}}$	$27.08~\frac{\text{nm}}{\text{fs}}$	$14.49~\frac{\text{nm}}{\text{fs}}$	$14.78~\frac{\text{nm}}{\text{fs}}$	$15.11~\frac{\text{nm}}{\text{fs}}$
step: $n_a = 3$	$3.34~\frac{\text{nm}}{\text{fs}}$	$3.58~\frac{\text{nm}}{\text{fs}}$	$7.21~\frac{\text{nm}}{\text{fs}}$			
step: $n_a = 4$	$1.56~\frac{\text{nm}}{\text{fs}}$	$6.91~\frac{\text{nm}}{\text{fs}}$	$6.30~\frac{\text{nm}}{\text{fs}}$			
step: $n_a = 5$	$6.74~\frac{\text{nm}}{\text{fs}}$	$6.10~\frac{\text{nm}}{9}2\text{fs}$				
step: $n_a = 6$	$5.91~\frac{\text{nm}}{\text{fs}}$					
**Fractions**	f_{1,n_1}	f_{2,n_2}	f_{3,n_3}	f_{4,n_4}	f_{5,n_5}	f_{6,n_6}
step: $n_a = 1$	0.8165	0.8165	0.8165	0.7848	0.7848	0.7848
step: $n_a = 2$	0.9360	0.9344	0.9373	0.9866	0.9861	0.9855
step: $n_a = 3$	0.9989	0.9988	0.9956			
step: $n_a = 4$	0.9998	0.9959	0.9976			
step: $n_a = 5$	0.9961	0.9978				
step: $n_a = 6$	0.9979					

Table S2: Total electronic eigenenergies of the states that represent the scattering signal at each step in the model of the Auger-Meitner decay of photoionized SF_6 as well as the corresponding kinetic energies, velocities, and fractions of the ejected electrons in the interaction region. The eigenenergies are given relative to the eigenenergy of the neutral molecule in its electronic ground state, *i.e.* $E_{a,1}$ is equal to the transition energy of the respective photoionization. The kinetic energies of the photoelectrons are furthermore calculated with a mean-photon energy of $E_{\text{ph}} \approx 9486.4$ eV. The fractions are computed according to Eq. (S43) with the delay densities obtained from the XTCAV data shown in Extended Data Figure 2. As discussed in the text, the values of f_{a,n_a} constitute a lower limit for the fraction of free electrons present in the interaction region when the second X-ray photon is scattered.

References

[1] E. Prince, *International Tables For Crystallography* (Published for the International Union of Crystallography by Kluwer Academic, 2004).

[2] M. Simmermacher, N. E. Henriksen, K. B. Møller, A. Moreno Carrascosa, and A. Kirrander, J. Chem. Phys. **151**, 174302 (2019).

[3] A. Moreno Carrascosa, H. Yong, D. L. Crittenden, P. M. Weber, and A. Kirrander, J. Chem. Theory Comput. **15**, 2836 (2019).

[4] M. Simmermacher, P. M. Weber, and A. Kirrander, in *Structural Dynamics with X-ray and Electron Scattering* (Royal Society of Chemistry, 2023), chap. 3, pp. 85–125.

[5] B. Stankus, H. Yong, J. Ruddock, L. Ma, A. M. Carrascosa, N. Goff, S. Boutet, X. Xu, N. Zotev, A. Kirrander, et al., Journal of Physics B: Atomic, Molecular and Optical Physics **53** (2020).

[6] J. Als-Nielsen and D. McMorrow, *Elements of Modern X-ray Physics* (John Wiley & Sons, Ltd, 2011).

[7] L. S. Bartell and R. M. Gavin, J. Am. Chem. Soc. **86**, 3493 (1964).

[8] G. Dixit, J. M. Slowik, and R. Santra, Phys. Rev. A **89**, 043409 (2014).

[9] L. Ma, H. Yong, J. D. Geiser, A. Moreno Carrascosa, N. Goff, and P. M. Weber, Structural Dynamics **7** (2020).

[10] Linac Coherent Light Source, SLAC National Accelerator Laboratory, *CXI components*, URL https://lcls.slac.stanford.edu/instruments/cxi/components.

[11] M. Rumi and J. W. Perry, Advances in Optics and Photonics **2** (2010).

[12] E. Fasshauer and L. B. Madsen, Phys. Rev. A **101**, 043414 (2020).

[13] L. Inhester, B. Oostenrijk, M. Patanen, E. Kokkonen, S. H. Southworth, C. Bostedt, O. Travnikova, T. Marchenko, S.-K. Son, R. Santra, et al., J. Phys. Chem. Lett. **9**, 1156 (2018).

[14] K. Faegri and O. Keski-Rahkonen, J. Electron Spectrosc. **11**, 275 (1977).

[15] L. Asplund, P. Kelfve, B. Blomster, H. Siegbahn, K. Siegbahn, R. L. Lozes, and U. I. Wahlgren, Phys. Scripta **16**, 273 (1977).

[16] O. Grånäs, A. Mocellin, E. S. Cardoso, F. Burmeister, C. Caleman, O. Björneholm, and A. N. de Brito, J. Phys. B: At. Mol. Opt. **53**, 244007 (2020).

[17] R. G. Albridge, K. Hamrin, G. Johansson, and A. Fahlman, Z. Phys. A: Hadrons Nucl. **209**, 419 (1968).

[18] J. H. Scofield, *Theoretical photoionization cross sections from 1 to 1500 kev*, UCRL-51326, United States, (1973).

[19] B. Schimmelpfennig, B. Nestmann, and S. Peyerimhoff, Journal of Electron Spectroscopy and Related Phenomena **74**, 173 (1995).

[20] H.-J. Werner, P. J. Knowles, G. Knizia, F. R. Manby, and M. Schütz, WIREs Comput. Mol. Sci. **2**, 242 (2012).

[21] H.-J. Werner, P. J. Knowles, G. Knizia, F. R. Manby, M. Schütz, P. Celani, W. Györffy, D. Kats, T. Korona, R. Lindh, et al., *Molpro, version 2015.1, a package of ab initio programs* (2015), see http://www.molpro.net.

[22] C. Hampel, K. A. Peterson, and H.-J. Werner, Chem. Phys. Lett. **190**, 1 (1992).

[23] J. Dunning, Thom H., J. Chem. Phys. **90**, 1007 (1989).

[24] R. A. Kendall, J. Dunning, Thom H., and R. J. Harrison, J. Chem. Phys. **96**, 6796 (1992).

[25] D. E. Woon and J. Dunning, Thom H., J. Chem. Phys. **98**, 1358 (1993).

[26] W. A. de Jong, R. J. Harrison, and D. A. Dixon, J. Chem. Phys. **114**, 48 (2001).

[27] D. Peng and K. Hirao, J. Chem. Phys. **130**, 044102 (2009).

[28] H.-J. Werner and P. J. Knowles, J. Chem. Phys. **82**, 5053 (1985).

[29] P. J. Knowles and H.-J. Werner, Chem. Phys. Lett. **115**, 259 (1985).

[30] H. J. A. Jensen, P. Jørgensen, and H. Ågren, J. Chem. Phys. **87**, 451 (1987).

[31] J. Wang and V. H. Smith Jr, Int. J. Quantum Chem. **52**, 1145 (1994).

[32] N. Zotev, A. Moreno Carrascosa, M. Simmermacher, and A. Kirrander, J. Chem. Theory Comput. **16**, 2594 (2020).

[33] J. P. Coe, A. Moreno Carrascosa, M. Simmermacher, A. Kirrander, and M. J. Paterson, J. Chem. Theory Comput. **18**, 6690 (2022).

[34] K. Zähringer, H.-D. Meyer, and L. S. Cederbaum, Phys. Rev. A **46**, 5643 (1992).
